# Structure–property study of cross-linked hydrocarbon/poly(ethylene oxide) electrolytes with superior conductivity and dendrite resistance†
†Electronic supplementary information (ESI) available: Synthetic procedures, characterization data, electrochemical impedance spectra and additional SEM images. See DOI: 10.1039/c6sc01813k



**DOI:** 10.1039/c6sc01813k

**Published:** 2016-07-19

**Authors:** Qi Zheng, Lin Ma, Rachna Khurana, Lynden A. Archer, Geoffrey W. Coates

**Affiliations:** a Department of Chemistry and Chemical Biology , Baker Laboratory , Cornell University , Ithaca , New York 14853 , USA . Email: coates@cornell.edu; b Department of Materials Science and Engineering , Cornell University , Bard Hall , Ithaca , New York 14853 , USA; c School of Chemical and Biomolecular Engineering , Cornell University , Olin Hall , Ithaca , New York 14853 , USA . Email: laa25@cornell.edu

## Abstract

Lithium dendrite growth is a fundamental problem that precludes the practical use of lithium metal batteries. Solid polymer electrolytes (SPEs) have been widely studied to resist the growth of lithium dendrites but the underlying mechanisms are still unclear. Most SPEs sacrifice high ionic conductivities for increased dendrite suppression performance by using components with high mechanical stiffness. We report a class of cross-linked hydrocarbon/poly(ethylene oxide) SPEs with both high ionic conductivities (approaching 1 × 10^–3^ S cm^–1^ at 25 °C) and superior dendrite suppression characteristics. A systematic structure–property study shows that the crystallinity of the hydrocarbon backbones plays a key role in regulating size and morphology of lithium dendrites, as well as the ability to suppress their growth.

## Abstract 2

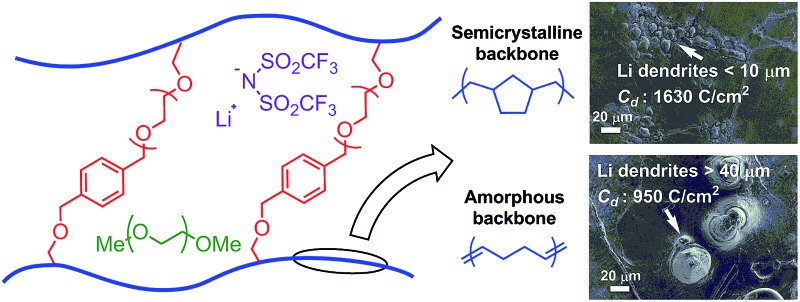
The influence of macromolecular composition on Li dendrite supression in solid polymer electrolytes was elucidated.

## Introduction

The possibility of replacing the lithiated graphitic carbon LiC_6_ anode in lithium-ion batteries with metallic lithium has been the subject of extensive research because metallic lithium has a specific capacity approximately 10 times that of conventional lithiated graphite (3860 mA h g^–1^ *vs.* 370 mA h g^–1^).^1,2^ A fundamental problem with metallic lithium anodes is their propensity to form rough, dendritic electrodeposits during cell recharge. While catastrophic cell failure due to dendrite-induced short circuits and the potential for thermal runaway are often cited as the main consequences of rough electrodeposition, an equally important problem is increased reactivity between electrodeposits and liquid electrolytes, which lowers cell efficiency and ultimately leads to premature failure over prolonged cycling.^1,3,4^ Many strategies have been developed to solve the problem of rough Li electrodeposition, including coating Li anodes with polymers,^5^ introducing additives into the electrolyte,^6–8^ inserting an interlayer between the anode and the electrolyte,^9^ and careful design of solid polymer electrolytes.^10–13^

**Fig. 1:**
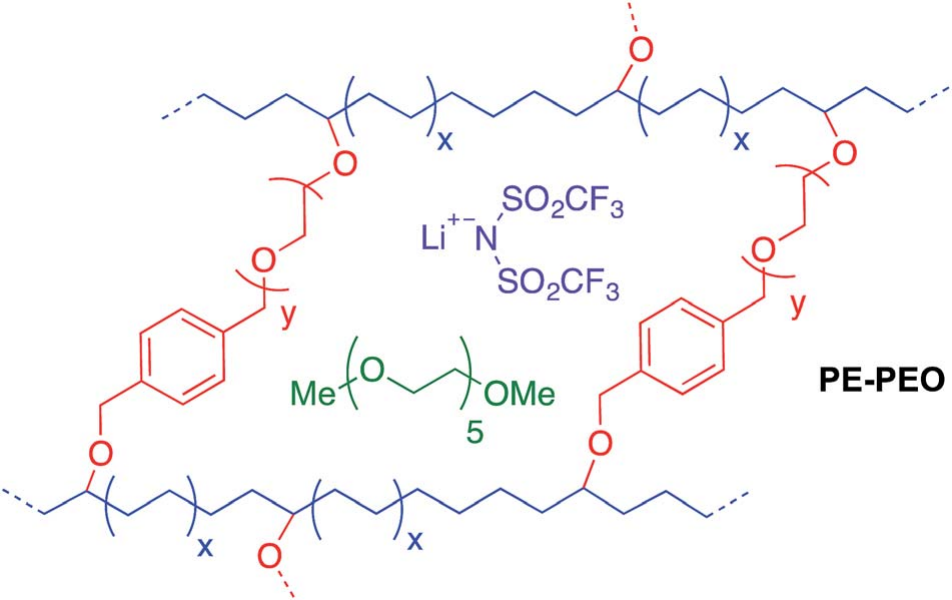
Schematic of cross-linked polyethylene/poly(ethylene oxide) (PE–PEO) solid polymer electrolyte (SPE).^10^

Among all these strategies, self-assembled nanostructured electrolyte architectures produced by block copolymers^12,13^ offer a particularly versatile platform for dendrite inhibition. Archer *et al.* reported a cross-linked material of hairy silica nanoparticles and poly(propylene oxide), which can be cycled for more than 1000 hours in a lithium symmetric cell at a current density (*J*) of 0.2 mA cm^–2^.^14^ A similar cross-linked material between polyhedral oligomeric silsesquioxane (POSS) and poly(ethylene glycol) was synthesized by Li and co-workers.^15^ This material has a *C*_d_ (total charge passed at cell failure in a galvanostatic cycling test) of 2800 C cm^–2^ at *J* = 0.3 mA cm^–2^. These two examples are believed to be the state-of-the-art of Li dendrite suppression for solid polymer electrolytes. Recently, Balsara and co-workers^11^ reported high shear moduli polystyrene-*block*-poly(ethylene oxide) (PS-*b*-PEO) polymers that exhibited decent dendrite growth resistance. This work validated the Monroe and Newman's model^16,17^ for dendrite growth inhibition. Their model predicts that a surface layer with high shear modulus (*G*′ > 7 GPa) can physically suppress the growth of lithium dendrites. Recently, our group^10^ developed a family of PE–PEO cross-linked SPEs (see Fig. 1) that showed exceptional Li dendrite suppression, which in some cases was one magnitude higher than that of PS-*b*-PEO as deduced by *C*_d_ in a galvanostatic cycling test. Significantly, the shear moduli of the best-performing cross-linked PE–PEO SPEs were three orders of magnitudes lower than that of PS-*b*-PEO (∼0.1 MPa compared with ∼0.1 GPa), which suggests that a high shear modulus is not essential for good Li dendrite resistance. Moreover, the ionic conductivity of these SPEs were two orders of magnitude higher than PS-*b*-PEO at room temperature, making them promising for practical use at ambient temperatures.

The role of the PE main chain with respect to the improved dendrite resistance of the PE–PEO cross-linked polymer is currently unclear. To investigate the effect of the physical properties of these polymer main chains on the exceptional overall Li dendrite resistance of the materials, we prepared a series of PEO cross-linked polymers with main chains comprising semicrystalline hydrogenated polynorbornene (hPNB) and amorphous, unsaturated polycyclooctadiene (PCOD). This composition allowed us to systematically vary the crystallinity of the materials and thereby the mechanical stiffness of the main chain. Although the Li–metal battery dendrite phenomenon is known for more than 50 years, none of the presented remedies are able to tackle both the safety issue as well as the low cycling performance. Despite the extensive research that has been performed on the fundamentals of dendrite growth,^4,16–18^ the underlying mechanisms are still not clear. In this work, our goal is to gain better understanding regarding the structure–property relationships of the polymer main chain on the Li dendrite resistance of the materials. The results of this study are reported herein.

## Results and discussion

The goal of the study was to determine how the physical properties of SPEs comprising hydrocarbon backbones with varied crystallinity compare with those of previously reported highly crystalline PE–PEO SPEs, and to systematically evaluate how these properties influence the capability of SPEs to retard dendrite growth. Atactic hPNB (melting temperature (*T*_m_): 143 °C) and PCOD (no *T*_m_) were selected because they are semicrystalline^19^ and amorphous, respectively. The comparison of PCOD, hPNB, and PE hydrocarbon chains permitted a complete study of the crystallinity effects of backbones in hydrocarbon/PEO cross-linked SPEs.

Cross-linked polymers with incorporated PEO segments are usually synthesized with UV irradiation^20^ or by reacting PEO with tri-isocyanates.^21^ However, these methods offer little structural control of the synthesized polymers. Building on our recent work on cross-linked alkaline anion-exchange membranes,^22^ we developed a tandem catalyst system with Grubbs' and Crabtree's catalysts. This system allows orthogonal catalysis of ring-opening metathesis polymerization to form polymers containing cross-linked PEO (Fig. 1) that can undergo subsequent olefin hydrogenation to tune the crystallinity of the hydrocarbon backbones.

**Fig. 2:**
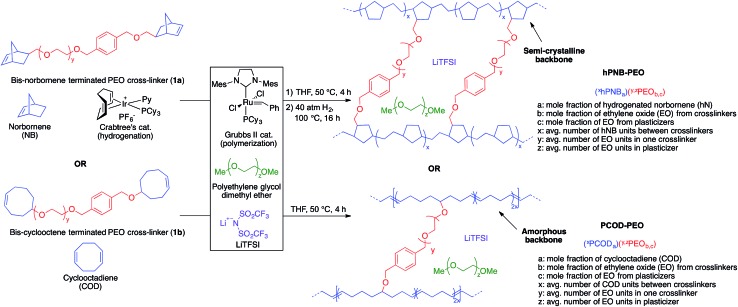
Hydrogenated polynorbornene–polyethylene oxide (hPNB–PEO) and polycyclooctadiene–polyethylene oxide (PCOD–PEO) cross-linked polymer electrolyte synthesis and nomenclature.

**Fig. 3:**
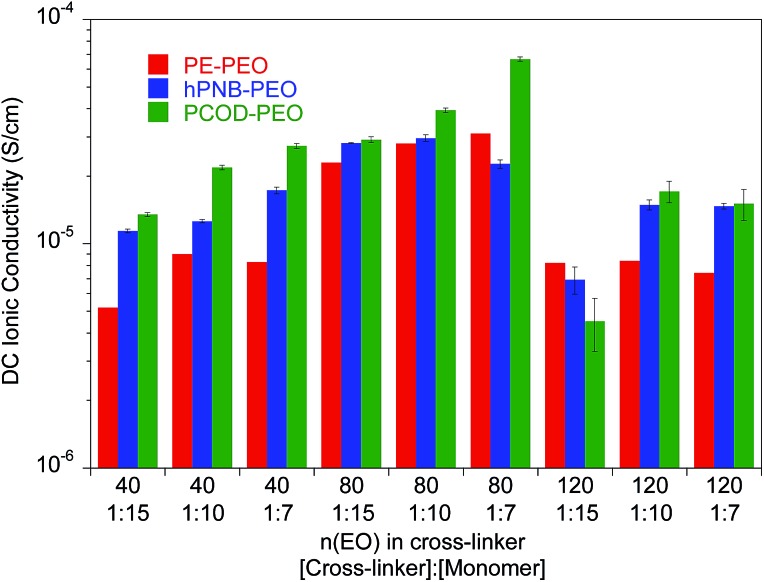
Ionic conductivity of unplasticized polymer electrolyte with different numbers of ethylene oxide (EO) units in the cross-linker and various [cross-linker] : [monomer] ratios at 25 °C. The data for PE–PEO SPEs are from ref. 10.

Norbornene- or cyclooctene-terminated PEO cross-linkers (**1a**, **1b**) were synthesized *via* anionic ring-opening polymerization of ethylene oxide (EO). Macromonomer **1a** was copolymerized with norbornene in a Teflon-coated mold with Grubbs' second-generation catalyst and lithium bis(trifluoromethane)sulfonimide (LiTFSI) as a lithium salt in THF (Fig. 2). Macromonomer **1b** was copolymerized with cyclooctadiene by using the same procedure. Translucent thin membranes were obtained after evaporating THF at 50 °C for 4 h. Membranes prepared from **1a** and norbornene were then hydrogenated with Crabtree's catalyst under 40 atm hydrogen gas at 100 °C for 16 h. The number of EO repeating units in the cross-linkers (*ca.* 40, 80, and 140 units *via* anionic ring-opening polymerization) and the [**1a**] : [NB] or [**1b**] : [COD] ratios (1 : 7, 1 : 10, 1 : 15 *via* ring-opening metathesis polymerization) were varied to prepare materials with a range of compositions. Nine different hPNB–PEO and PCOD–PEO SPEs were made (see ESI for details†). The ionic conductivities of these SPEs at room temperature were quantified from the plateau conductivity in dielectric measurements and compared with those of the analogous PE–PEO SPEs (Fig. 3).

As reported earlier for the PE–PEO SPEs, the number of EO units in the cross-linker played a key role in determining the ionic conductivity for hPNB–PEO and PCOD–PEO SPEs as well. Among the tested SPEs, electrolytes containing 80 EO cross-linker units showed maximum conductivity. In the polymers with 40 EO cross-linker units, the crystallinity of the PEO segments was completely suppressed by either the cross-linking structure or the backbones (no *T*_m_ observed; see ESI†). Segmental movements of the chain were also significantly reduced, which led to relatively low conductivity. With 140 EO units, PEO segments resumed being crystalline, hampering the room-temperature conductivity. Therefore, cross-linkers containing 80 EO units lie between the two extremes and give the highest conductivities.

Ionic conductivity of the SPEs showed an interesting dependence on the crystallinity of the polymer backbones when the cross-linking density was varied. For crystalline backbones such as PE and hPNB, no substantial changes were observed when the spacing of the cross-linkers was changed. However, for the amorphous PCOD backbone, conductivity increased with closer cross-linker spacing (higher [**1b**] : [COD] ratio). We hypothesize that crystalline backbones limit the segmental movement of PEO segments, whereas an amorphous backbone facilitates motion. Closer spacing of the cross-linkers would not improve Li-ion transport in frozen PEO chains but would greatly improve Li interchain transport in movable PEO chains. The overall conductivity was found to be inversely related to the degree of backbone crystallinity.

To improve the conductivity further, we used various amounts of poly(ethylene glycol) dimethyl ether (**PEG**; molecular weight, 275 Da; 16, 24, 32, 40 wt%) to plasticize the SPEs with approximately 80 EO repeating cross-linker units and 1 : 15 for [**1a**] : [NB] or [**1b**] : [COD] concentration ratios. The number of EO repeating cross-linker units and [**1a**] : [NB] or [**1b**] : [COD] concentration ratios were chosen to be consistent with our previous studies^10^ so that we can make comparisons among different systems. The compositions and thermal properties of the plasticized samples are reported in Table 1.

**Table 1:** Compositions and Vogel–Tammann–Fulcher fitting parameters of plasticized hydrogenated polynorbornene–polyethylene oxide (hPNB–PEO) and polycyclooctadiene–polyethylene oxide (PCOD–PEO) solid polymer electrolytes (SPEs)

Entry	Plasticized SPE^*a*^	Weight% of **PEG** ^*b*^	hPNB segments^*c*^		PEO segments^*c*^				*E*_a_ (kJ mol^–1^)	*A* (S cm^–1^)
			*T*_m_ ^*d*^ (°C)	Δ*H*_fus_ ^*d*^ (J g^–1^)	*T*_g_ ^*d*^ (°C)	*T*_c_ ^*d*^ (°C)	*T*_m_ ^*d*^ (°C)	Δ*H*_fus_ ^*d*^ (J g^–1^)		
1	(^8.5^hPNB_0.25_)(^88,0^PEO_0.75,0_)	0	92	2.8	–48	–6	32	27.9	10.0	0.48
2	(^8.5^hPNB_0.14_)(^88,5^PEO_0.64,0.22_)	16	101	4.2	–60	–27	28	42.7	9.7	0.52
3	(^8.5^hPNB_0.13_)(^88,5^PEO_0.57,0.30_)	24	95	3.6	–64	–31	25	35.9	9.6	0.71
4	(^8.5^hPNB_0.11_)(^88,5^PEO_0.46,0.43_)	32	94	3.0	–69	–40	22	31.9	8.8	0.71
5	(^8.5^hPNB_0.09_)(^88,5^PEO_0.38,0.54_)	40	89	3.0	–70	–35	20	34.1	8.6	1.01
6	(^8.5^PCOD_0.17_)(^75,0^PEO_0.83,0_)	0	n.d.^*e*^	n.d.^*e*^	–41	n.d.^*e*^	n.d.^*e*^	n.d.^*e*^	8.6	0.20
7	(^8.5^PCOD_0.13_)(^75,5^PEO_0.66,0.21_)	16	n.d.^*e*^	n.d.^*e*^	–46	n.d.^*e*^	n.d.^*e*^	n.d.^*e*^	8.1	0.24
8	(^8.5^PCOD_0.11_)(^75,5^PEO_0.57,0.32_)	24	n.d.^*e*^	n.d.^*e*^	–50	n.d.^*e*^	n.d.^*e*^	n.d.^*e*^	8.2	0.40
9	(^8.5^PCOD_0.10_)(^75,5^PEO_0.48,0.43_)	32	n.d.^*e*^	n.d.^*e*^	–51	n.d.^*e*^	n.d.^*e*^	n.d.^*e*^	7.8	0.42
10	(^8.5^PCOD_0.08_)(^75,5^PEO_0.39,0.53_)	40	n.d.^*e*^	n.d.^*e*^	–55	n.d.^*e*^	n.d.^*e*^	n.d.^*e*^	8.1	0.60

^*a*^See Fig. 2 for nomenclatures. All films have [EO] : [Li] compositions of 20 : 1, where EO indicates ethylene oxide units in the cross-linker.

^*b*^Wt% of **PEG** plasticizer = (mass of **PEG**)/[(mass of **PEG**) + (mass of PEO from cross-linker) + (mass of norbornene) + (mass of LiTFSI)] × 100.

^*c*^Thermal property data for hPNB and PEO segments.

^*d*^Glass transition temperature (*T*_g_), cold crystallization temperature (*T*_c_), melting temperature (*T*_m_), and enthalpy of fusion (Δ*H*_fus_) were determined with differential scanning calorimetry from the second heat cycle.

^*e*^Not detected.

**Fig. 4:**
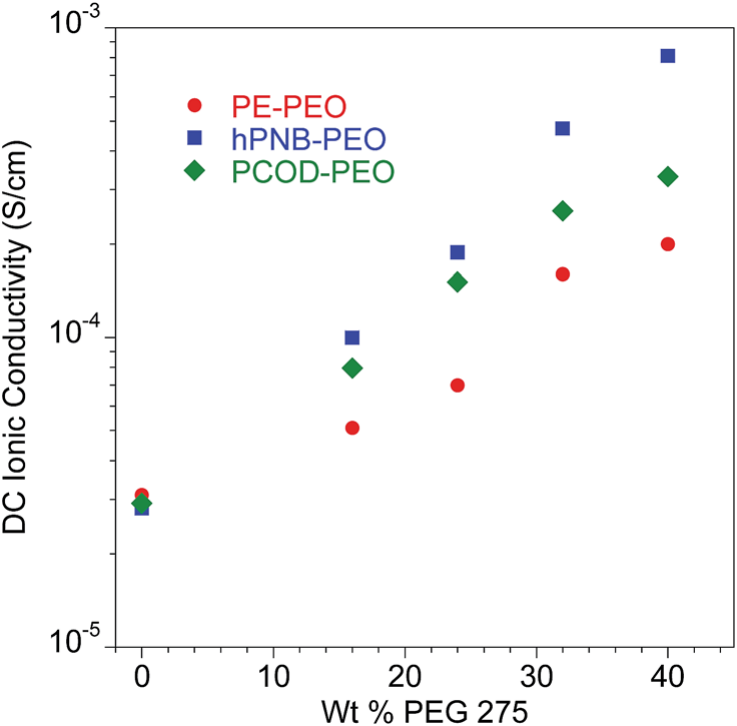
Ionic conductivity of plasticized polymer electrolyte as a function of wt% of **PEG** 275 at 25 °C. All membranes had approximately 80 EO cross-linker units (70 for PE–PEO,^10^ 88 for hPNB–PEO, and 75 for PCOD–PEO), [**1a**] : [NB] or [**1b**] : [COD] ratios of 1 : 15, and [EO] : [Li] compositions of 20 : 1. Error bars are smaller than the size of the data points. The data for PE–PEO SPEs are from ref. 10.

The addition of plasticizers greatly decreased the glass transition temperature (*T*_g_) of PEO segments in the SPEs. For hPNB–PEO, *T*_g_ dropped from –48 °C (Table 1, entry 1) to –70 °C in the presence of 40 wt% **PEG** (entry 5). A decrease was also observed for PCOD–PEO, for which *T*_g_ changed from –41 °C (0 wt%, entry 6) to –55 °C (40 wt%, entry 10). Significant decreases were also observed in cold crystallization temperature, and *T*_m_ for PEO segments, indicating that the crystallinity of the cross-linkers was greatly suppressed by **PEG** oligomers. However, *T*_m_s of the hPNB segments were not affected significantly by plasticizers. The relative percent crystallinity is in the range of 4.3% to 6.4% (Δ*H*_fus_ for pure hPNB is 65.4 J g^–1^ (ref. 19)), which is slightly lower than what we observed in PE–PEO^10^ (5.1% to 6.9%). The absolute value of Δ*H*_fus_ is lower in hPNB–PEO than in PE–PEO, which confirms that the hPNB–PEO has a lower crystallinity compared to PE–PEO. Fig. 4 reports the room temperature ionic conductivities of plasticized cross-linked hPNB–PEO, PCOD–PEO, and PE–PEO systems. Ionic conductivity increased with increasing **PEG** loading. Notably, the conductivity of 40 wt% plasticized hPNB–PEO reached 8.1 × 10^–4^ S cm^–1^, almost one order of magnitude higher than the minimum conductivity (1 × 10^–4^ S cm^–1^) required to use SPEs in commercial batteries at ambient temperature.

Both plasticized hPNB–PEO and PCOD–PEO SPEs exhibited higher ionic conductivities than those of PE–PEO SPEs at the same **PEG** loading. We attribute this increase to the lower crystallinity of the backbones, which allows better segmental movement of PEO chains, and thus faster Li ion transport. Variable-temperature ionic conductivities from 10 °C to 100 °C with an increment of 15 °C were also measured for entries 1–10 in Table 1 (see ESI for details†). The data can be well-described with the Vogel–Tammann–Fulcher equation (eqn (1)), which is widely used to describe the temperature dependence of ionic conductivity for polymers:^23^
1
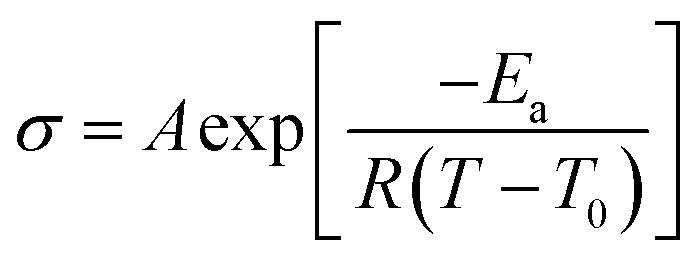
where *σ* is the ionic conductivity, *A* is the prefactor, *E*_a_ is the activation energy, *R* is the gas content, and *T*_0_ is the ideal *T*_g_, which was selected to be 50 K below the experimental *T*_g_ values of SPEs. The Vogel–Tammann–Fulcher parameters are summarized in Table 1. The activation energy dropped with increasing wt% of **PEG** in the hPNB–PEO system, but no clear trends were observed in the PCOD–PEO system. The decrease in activation energy of the former system is expected because the plasticizer helps lower the energy barrier for Li ion conduction. The prefactor *A* increases with increasing wt% of **PEG** in both the hPNB–PEO and the PCOD–PEO systems. *A* is proportional to the number of charge carriers. The higher wt% of **PEG** provides more solvation centers, producing the increase in *A*.

To investigate the lifetime of lithium–metal-based batteries (LMB), we carried out galvanostatic lithium plate/strip electrochemical cycling measurements in symmetric Li/SPE/Li cells with a 3 h lithium plating followed by a 3 h lithium stripping at a current density (*J*) of 0.26 mA cm^–2^ and 90 °C. The 3 h period mimics the charge/discharge profiles of typical cells and ensures that the quantities of lithium transported during each cycle are sufficient to create dendrites large enough to short-circuit the cell.^10,24^ The temperature was chosen to be consistent with earlier experiments. This temperature is above the *T*_m_s of PEO segments so that a good conductivity can be achieved, but below the *T*_m_s of hydrocarbon backbones in PE–PEO and hPNB–PEO so that the crystallinity was maintained. Under these conditions, the conductivities of the hPNB–PEO and PCOD–PEO are nearly identical, allowing us to remove the trivial influence of electrolyte conductivity on dendrite suppression features of the copolymers. The effects of the various backbones in the three SPEs on suppressing dendrite growth was quantified by the total charge passed, *C*_d_, at the time of cell failure as a result of dendrite-induced short-circuits. A *C*_d_ value of 1630 C cm^–2^ was observed for hPNB–PEO, which is similar to that for PE–PEO (1790 C cm^–2^) reported in our earlier paper.^10^ Thus, lithium dendrite resistance is not significantly changed when semicrystalline hPNB is used instead of crystalline PE, but the conductivity is three times higher than that of PE–PEO at ambient temperature. When the crystallinity is further suppressed by modifying the backbone with PCOD, *C*_d_ decreases to approximately half that of PE–PEO.

**Fig. 5:**
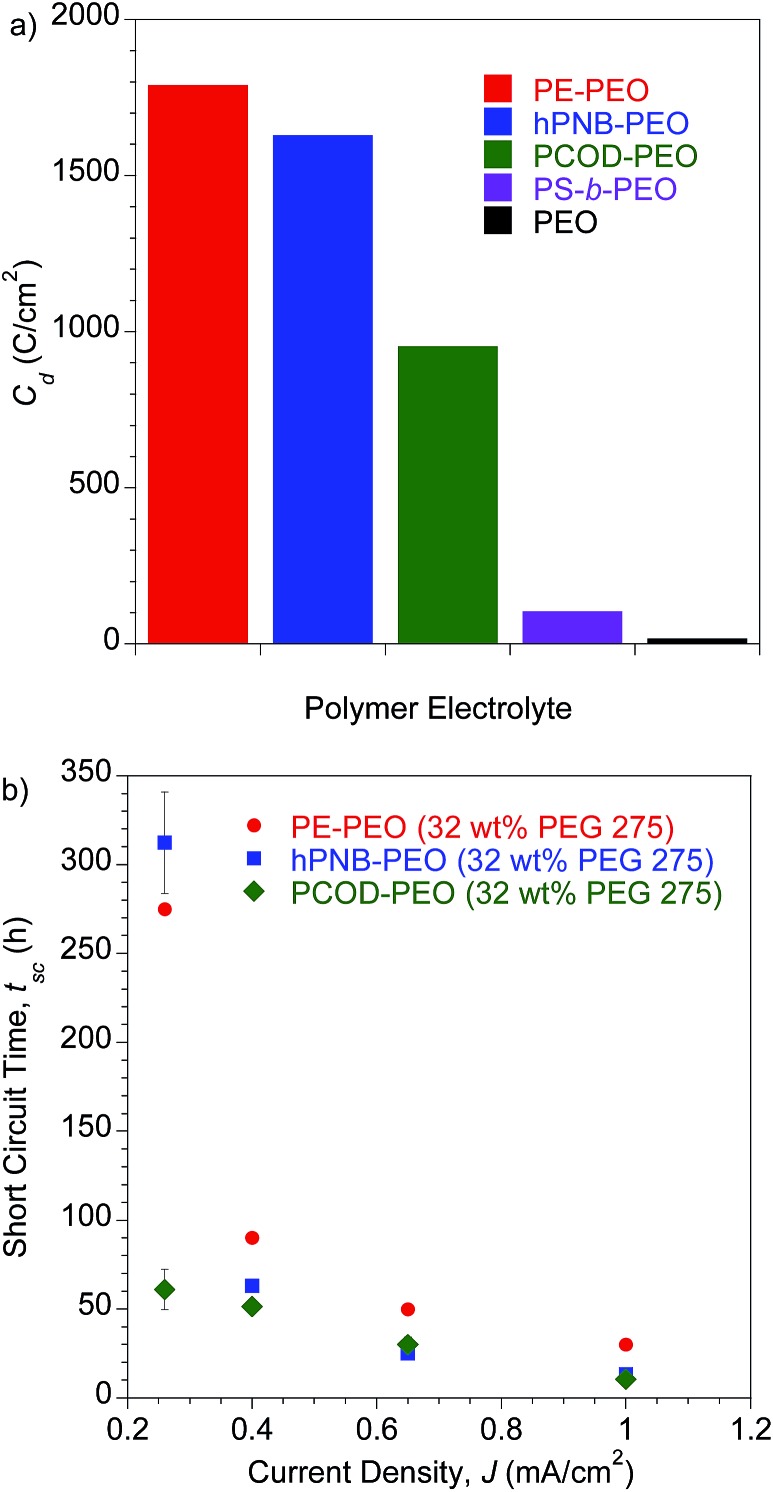
(a) Galvanostatic cycling tests. Plot of *C*_d_ for polymer electrolytes. All samples were tested with a current density (*J*) of 0.26 mA cm^–2^ at 90 °C. Error bars are 33 h for hPNB–PEO and 67 h for PCOD–PEO. PE–PEO, PS-*b*-PEO, and PEO values are from the literature.^10,11^ (b) Galvanostatic polarization tests. Plot of short-circuit time (*t*_sc_) as a function of *J* at 90 °C. All membranes were plasticized with 32 wt% **PEG** 275 and had approximately 80 EO cross-linker units (70 for PE–PEO,^10^ 88 for hPNB–PEO, and 75 for PCOD–PEO), [**1a**] : [NB] or [**1b**] : [COD] ratios of 1 : 15, and [EO] : [Li] composition of 20 : 1.

A more aggressive galvanostatic polarization procedure was used to further characterize lithium electrodeposition and LMB cell failure in symmetric lithium cells polarized at a fixed *J*. The short-circuit time (*t*_sc_) was defined as the time at which a sudden voltage drop occurred (see Fig. S10†).^25^ The *t*_sc_ values for the crosslinked SPEs were measured at variable current densities (0.26–1.0 mA cm^–2^) at 90 °C, and the results are shown in Fig. 5b. Duplicate measurements were performed for each sample at each specified *J* value. Consistent with the findings in lithium plate/strip cycling measurement, the *t*_sc_ values of these cross-linked SPEs with hydrocarbon backbones were significantly higher than those of all other SPEs reported to date. PE–PEO had a *t*_sc_ higher than that of hPNB–PEO, and PCOD–PEO had the lowest *t*_sc_. Unlike in galvanostatic cycling measurements, where hPNB–PEO had a comparable *C*_d_ value to PE–PEO, hPNB–PEO had significantly lower *t*_sc_ than PE–PEO in galvanostatic polarization measurements, especially at high current densities. We attribute this phenomenon to the more aggressive conditions of galvanostatic polarization procedures.

**Fig. 6:**
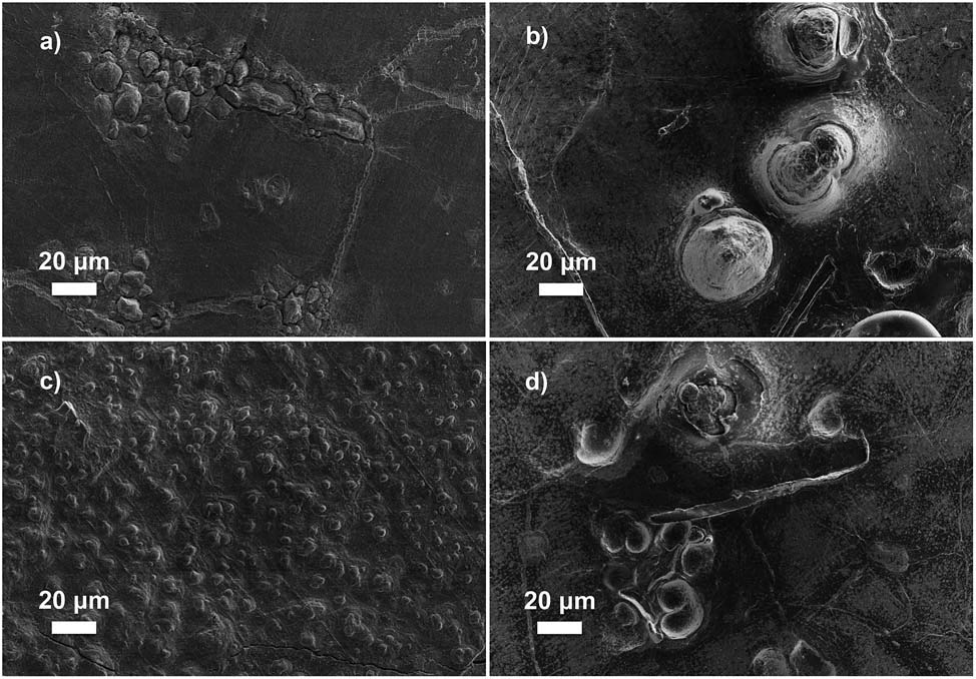
Scanning electron microscopy images of a short-circuited Li anode after galvanostatic (a) polarization (0.40 mA cm^–2^) and (c) cycling (0.26 mA cm^–2^) test with hPNB–PEO electrolyte (plasticized with 32 wt% **PEG** 275) and (b) polarization (0.40 mA cm^–2^) and (d) cycling (0.26 mA cm^–2^) test with PCOD–PEO electrolyte (plasticized with 32 wt% **PEG** 275).

The data for galvanostatic tests indicates that the polymer crystallinity plays a significant role in delaying the dendrite growth. We hypothesize that backbones with greater crystallinity create better-defined nanopore structures in the networks to provide enhanced resistance to the growth of micron-scale lithium dendrites, which increases *C*_d_ values (Fig. 5a).^26^ This hypothesis was further examined through post-mortem scanning electron microscopy to characterize the lithium metal anode and observe the morphology of the lithium surface after short-circuiting (Fig. 6). Fig. 6a and c show the morphology of the lithium deposition in hPNB–PEO after a short-circuiting event during polarization and cycling measurements, respectively. Fig. 6b and d display the SEM pictures of PCOD–PEO, after a short-circuiting event during galvanostatic polarization and cycling measurements respectively. Small ramified electrodeposits (<10 µm) were observed for hPNB–PEO, whereas much larger protrusions (>40 µm) were observed for PCOD–PEO. These results may be due to the higher crystallinity in hPNB–PEO compared with that of PCOD–PEO. The dendrites grow by creating splay-like openings in the softer PCOD–PEO membrane to produce large structures, whereas the crystallites in hPNB–PEO produce materials with stronger main-chain domains and hence higher splay resistance, permitting only smaller dendrites to penetrate the membrane.

**Fig. 7:**
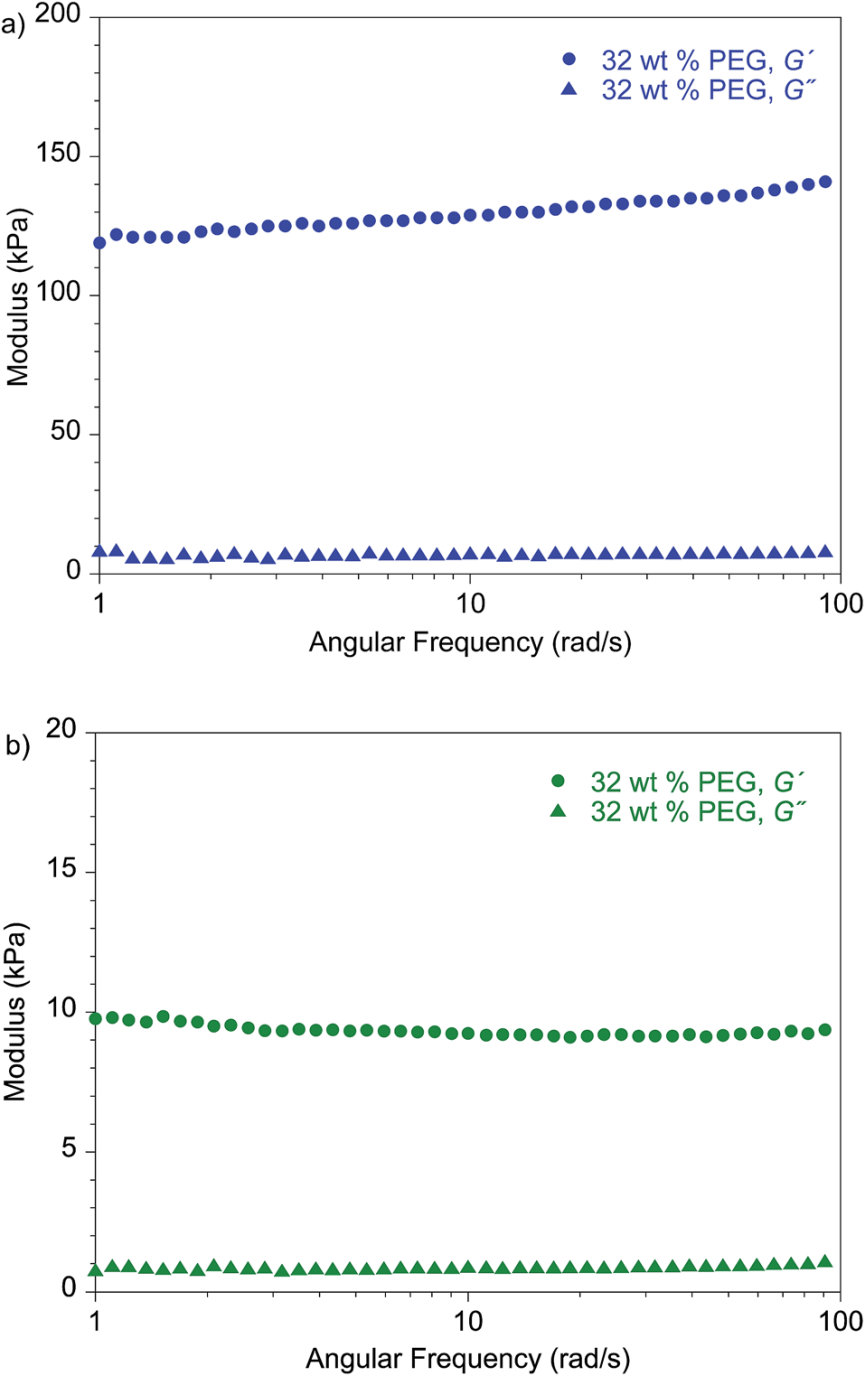
Frequency-dependent dynamic shear measurements on (a) hPNB–PEO and (b) PCOD–PEO electrolytes. All membranes were plasticized with 32 wt% **PEG** 275 and had approximately 80 EO cross-linker units (88 for hPNB–PEO and 75 for PCOD–PEO), [**1a**] : [NB] or [**1b**] : [COD] ratios of 1 : 15, and [EO] : [Li] compositions of 20 : 1.

Notably, the lithium dendrite resistance of the modified PEO system with different backbones is still much higher than those reported for other SPEs at a comparable current density: PS-*b*-PEO^11^ has a *C*_d_ value of 105 C cm^–2^, and the *C*_d_ of standard PEO^10^ (molecular weight, 900 kDa) is ∼20 C cm^–2^. Fig. 7 shows the shear moduli for the respective materials. The moduli of the **PEG**-plasticized hPNB–PEO and PCOD–PEO both exhibit only weak dependence on frequency, and the elastic shear modulus *G*′ is at least an order of magnitude greater than the loss modulus *G*″. Both observations are consistent with expectations of materials with network structures, with cross-link spacing of approximately 4.8 nm for hPNB–PEO and 11.3 nm for PCOD–PEO. As in our previous studies of PE–PEO networks, an order of magnitude higher *C*_d_ values are obtained for materials with rather modest shear moduli (*G*′ ∼ 10^5^ Pa at 90 °C) in both PE–PEO and hPNB–PEO. Even PCOD–PEO, which has a *G*′ one order of magnitude lower than that of hPNB–PEO, has a *C*_d_ that is much higher than those of most reported systems.^11^ However, the earlier conjecture that the *C*_d_ of hPNB–PEO is higher than that of PCOD–PEO because the main chain is stiffer and gives the material greater resistance to dendrite penetration is supported by the higher modulus of the former material, meaning that the mechanical properties of the materials do matter, but the effect in these networks is likely more complex than that captured in the solid separator model studied by Monroe and Newman.^16,17^ The systems developed in our lab produce very promising hydrocarbon/PEO cross-linked SPEs with high lithium dendrite resistance. In particular, hPNB–PEO is of special interest for Li metal based high energy density batteries due to both high lithium dendrite resistance and high conductivity.

## Conclusions

We developed two new hydrocarbon/PEO cross-linked SPEs: hPNB–PEO and PCOD–PEO. hPNB–PEO shows exceptionally high ionic conductivity at room temperature (approaching 10^–3^ S cm^–1^) and significant lithium dendrite suppression. PCOD–PEO retains half of the hPNB–PEO *C*_d_ value even though its shear modulus is an order of magnitude lower than that of PE–PEO. This result suggests that a high shear modulus is not essential for good lithium dendrite suppression. Our systematic comparison of hydrocarbon backbones suggests that crystallinity plays a central role in the size and morphology of lithium dendrites. We believe that these hydrocarbon/PEO cross-linked systems with high conductivity and good lithium dendrite resistance are promising candidate SPEs for future batteries that use Li metal anodes. We are currently performing battery device testing and morphology studies to further characterize the microstructures of these materials.
