# Supplementary material for: Structure–property study of cross-linked hydrocarbon/poly(ethylene oxide) electrolytes with superior conductivity and dendrite resistance
Source: Chem Sci. 2016 Jul 19;7(11):6832–8. doi: 10.1039/c6sc01813k (PMC5356005; doi:10.1039/c6sc01813k)
Supplement: Supplementary file 1 [file SC-007-C6SC01813K-s001.pdf]

Electronic Supplementary Material (ESI) for Chemical Science.
This journal is © The Royal Society of Chemistry 2016

Supporting Information

Structure-property study of cross-linked hydrocarbon-poly(ethylene oxide) electrolyte with superior conductivity and dendrite resistance

Qi Zheng,[a] Lin Ma,[b] Rachna Khurana,[a,†] Lynden A. Archer,[*,c] and Geoffrey W. Coates[*,a]

[a] Department of Chemistry and Chemical Biology, Baker Laboratory, Cornell University, Ithaca, New York 14853, United States

[b] Department of Materials Science and Engineering, Bard Hall, Cornell University, Ithaca, New York 14853, United States

[c] School of Chemical and Biomolecular Engineering, Olin Hall, Cornell University, Ithaca, New York 14853, United States

† Present address: 3M Company, Saint Paul, Minnesota 55144, United States

1. General Considerations

All air and water sensitive reactions were carried out under dry nitrogen conditions. ^{1}H NMR spectra were collected on a Varian INOVA 500MHz spectrometer and referenced with residue non-deuterated solvent shifts ($CHCl_3$ = 7.26 ppm). ^{13}C NMR spectra were collected on a Varian INOVA (^{13}C, 125 MHz) spectrometer and referenced to chloroform (δ 77.23 ppm). High resolution mass spectrometry (DART-HRMS) analyses were performed on a Thermo Scientific Exactive Orbitrap MS system equipped with an Ion Sense DART ion source. Matrix-assisted laser desorption/ionization time-of-flight mass spectrometry (MALDI-TOF MS) analyses were performed on a Waters MALDI Micro MX using dithranol as the matrix.

Gel permeation chromatography (GPC) analyses were carried out using an Agilent PL-GPC 50 integrated system, equipped with UV and refractive index detectors, and 2 PL gel Mini-MIX C columns (5 micron, 4.6 mm ID). The GPC columns were

eluted with tetrahydrofuran at 30 °C at 0.3 mL/min and were calibrated with monodisperse polystyrene standards. Differential scanning calorimetry (DSC) analyses of polymer samples were performed on a TA Instruments Q1000 instrument equipped with liquid nitrogen cooling system. Polymer samples were made in aluminum pans and heated under nitrogen from −100 °C to 180 °C at a rate of 10 °C per minute and then cooled to −100 °C at a rate of 10 °C per minute, followed heating to 180 °C at a rate of 10 °C per minute. The glass transition temperature (T_g) and the melting temperature (T_m) were recorded from the second heating run.

The thickness of the cross-linked solid polymer electrolytes for all measurement purposes was measured with a Marathon CO030025 electronic digital micrometer. The conductivity data of the polymer electrolytes was obtained over a range of frequency (0.1 to 3×10^6 Hz) and temperature (10 °C to 100 °C) using a Novocontrol Dielectric Broadband Spectrometer fitted with a Quatro temperature control system. Conductivity measurements were performed using blocking/solid polymer electrolyte (SPE)/blocking cell orientation, using gold plated stainless steel electrodes.

2. Materials

Sodium hydride (95%), bicyclo[2.2.1]hept-5-ene-2-carboxaldehyde (95%), sodium borohydride, benzyl bromide, dithranol, Grubbs' 2nd generation catalyst and Crabtree's catalyst were purchased from Sigma-Aldrich and used as received. Bis(trifluoromethane)sulfonimide lithium salt, LiTFSI (99.95% trace metals basis), was purchased from Sigma-Aldrich and dried in vacuo at 90 °C for 24 h and transferred directly into the glove box. Norbornene (99%) and 1,5-cyclooctadiene (≥99%) were purchased from Sigma-Aldrich and dried over calcium hydride at 60 °C for 2 days and

then distilled and transferred into the glove box. Ethylene oxide was purchased from Sigma-Aldrich and dried over *n*-BuLi for 1h before use. Poly(ethylene glycol) dimethyl ether, PEG275 (M_n (NMR) = 275 Da; M_n (Sigma-Aldrich label) = 250 Da) was bought from Sigma-Aldrich, dried over activated 3 Å sieves for 48 hours, and degassed by three freeze-pump-thaw cycles before use. Dibromo-*p*-xylene (97%) was purchased from Alfa Aesar and used as received. Sodium hydroxide and sodium chloride were purchased from Mallinckrodt and used as received. HPLC grade tetrahydrofuran was purchased from Fischer Scientific and dried over an alumina column and degassed by three freeze-pump-thaw cycles before use. Chloroform was dried over P_2O_5 and distilled prior to use. Hydrogen (99.99%) was purchased from Airgas. $CDCl_3$ was purchased from Cambridge Isotope Laboratories (CIL) and used as received. Bis-cyclooctene terminated PEO cross-linker (**1b**) was synthesized as reported.[1] Potassium naphthalenide in THF was prepared from naphthalene and potassium at a concentration of 0.31 M (titrated with a standard benzoic acid solution until a persistent green color was observed as an end-point of the titration) and degassed by three freeze pump thaw cycles before use.

3. Synthesis of PEO Crosslinkers

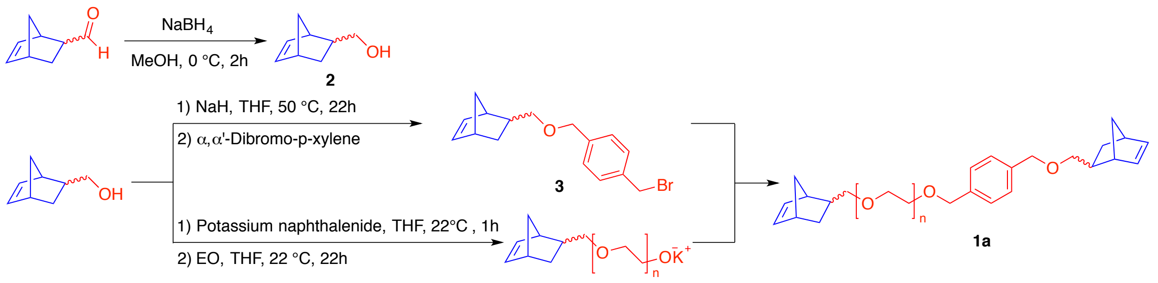

Scheme S1. General scheme for the synthesis of crosslinker **1a**.

3.1 Preparation of endo/exo-bicyclo[2.2.1]hept-5-en-2-ylmethanol (2)

Following a literature procedure,[2] to a solution of bicyclo[2.2.1]hept-5-ene-2-

carboxaldehyde (mixture of *endo* (67%) and *exo* (33%), 5.15 g, 42.2 mmol) in methanol (30 mL) was added dropwise a suspension of sodium borohydride (0.80 g, 21 mmol) in 2 M aqueous NaOH solution (20 mL) at 0 °C. The mixture was stirred for 2 h. The pH was then brought to 6 with 30 wt % H_2SO_4. Methanol was removed under vacuum and the residue was extracted with Et_2O (50 mL·3). The organic layer was washed with saturated $NaHCO_3$ (50 mL·3) and dried over anhydrous Na_2SO_4. Solvent was removed under vacuum and the residue was further purified by vacuum distillation (0.25 torr, 58 °C). The product was a viscous yellow oil (3.22 g, 61%). **^{1}H NMR** (500 MHz, $CDCl_3$) *endo*: δ 6.16 – 6.13 (m, 1H), 5.97 – 5.94 (m, 1H), 3.43 – 3.36 (m, 1H), 3.29 – 3.21 (m, 1H), 2.93 (br s, 1H), 2.81 (br s, 1H), 2.33 – 2.24 (m, 1H), 1.85 – 1.78 (m, 1H), 1.47 – 1.41 (m, 1H), 1.36 – 1.21 (m, 1H), 0.55 – 0.49 (m, 1H); *exo*: δ 6.12 – 6.05 (m, 2H), 3.74 – 3.67 (m, 1H), 3.58 – 3.50 (m, 1H), 2.81 (br s, 1H), 2.75 (br s, 1H), 1.65 – 1.57 (m, 1H), 1.36 – 1.21 (m, 3H), 1.14 – 1.08 (m, 1H). **^{13}C NMR** (126 MHz, $CDCl_3$) δ 137.58, 136.91, 136.58, 132.25, 67.65, 66.65, 49.66, 45.09, 43.70, 43.39, 42.33, 41.99, 41.81, 41.64, 29.64, 28.90. **HRMS** (DART) *m/z* calculated for $C_8H_{13}O^+$ $[M + H]^+$ 125.09609, found 125.09625.

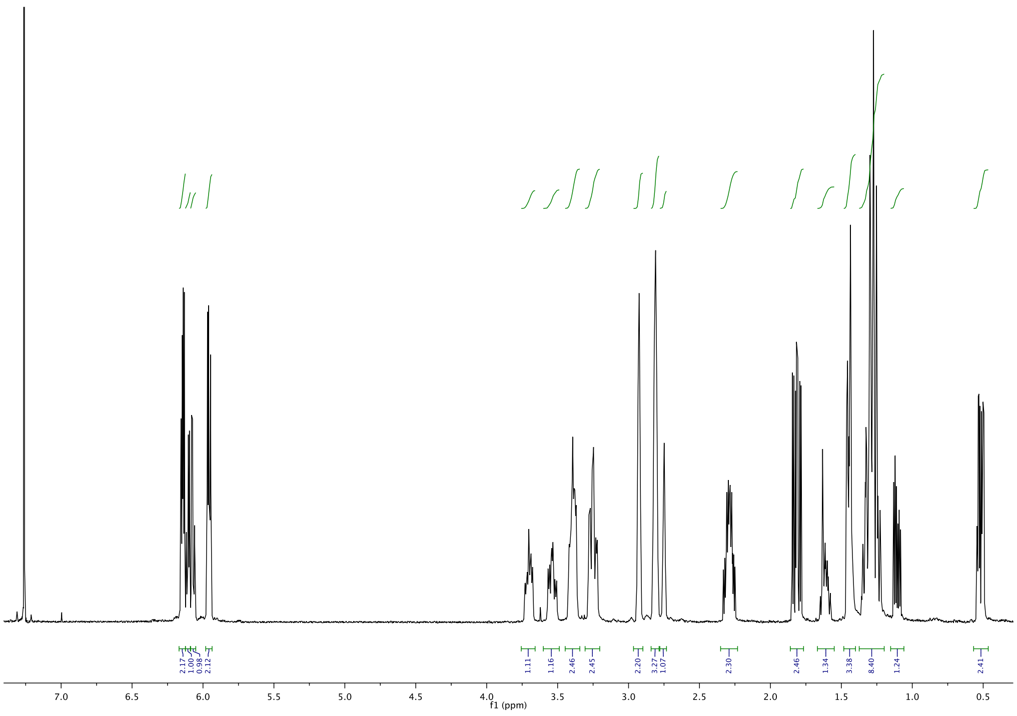

Figure S1. ^{1}H NMR spectrum of **2** in $CDCl_3$.

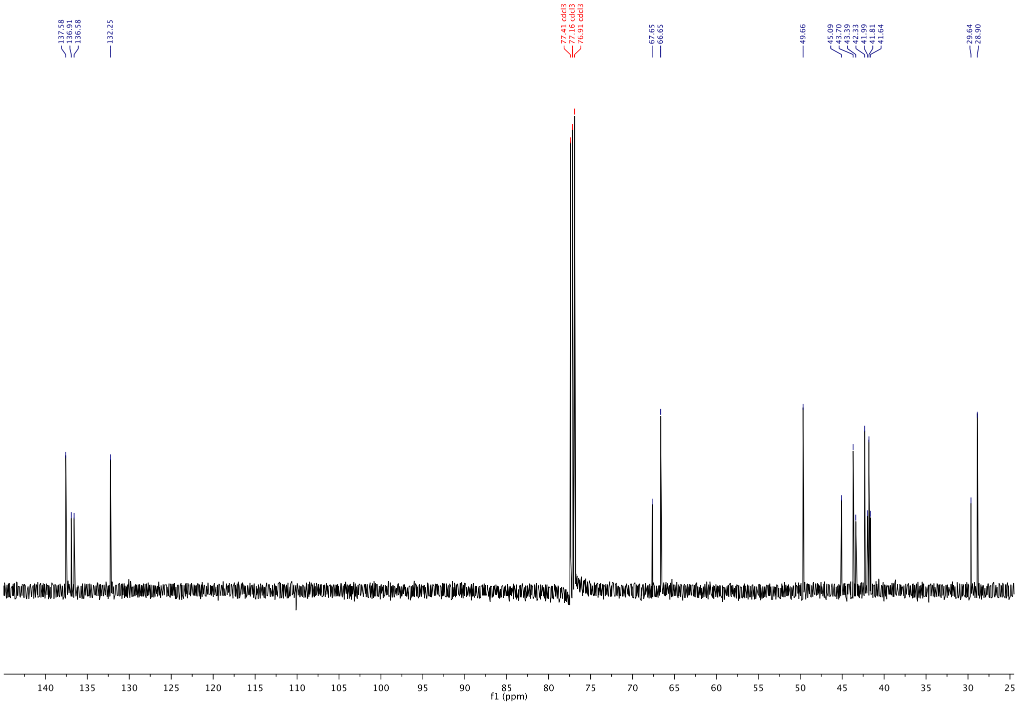

Figure S2. ^{13}C NMR spectrum of **2** in $CDCl_3$.

3.2 Preparation of endo/exo-5-(((4-(bromomethyl)benzyl)oxy)methyl)bicyclo[2.2.1]hept-2-ene (3)

To a suspension of NaH (581 mg, 24.2 mmol) in anhydrous THF (50 mL) was added dropwise a solution of **2** (2.00 g, 16.1 mmol) in anhydrous THF (20 mL). The mixture was heated to 50 °C under N_2 for 22 h. This solution was cooled to room temperature and cannula transferred to a solution of α,α'-dibromo-*p*-xylene (6.39 g, 24.2 mmol) in anhydrous THF (50 mL) under N_2. The solution turned to bright yellow with precipitation of salts. The solution was stirred at 22 °C for 16 h and quenched with minimum amount of ethanol. The solution was filtered and the filtrate was concentrated to yield colorless oil. Hexanes (~150 mL) were added to the residue oil and the solution was kept at 0 °C to recrystallize out excess dibromo-*p*-xylene. Dibromo-*p*-xylene was removed by filtration and the filtrate was concentrated and purified by column chromatography on silica using 1:1 CH_2Cl_2/hexanes. The product was isolated as a colorless oil (2.74 g, 55%). **^{1}H NMR** (500 MHz, $CDCl_3$) δ 7.40 – 7.29 (m, 4H), 6.13 – 5.86 (m, 2H), 4.53 – 4.41 (m, 4H), 3.55 – 3.50 (m, 1H *exo*), 3.40 – 3.34 (m, 1H *exo*), 3.23 – 3.17 (m, 1H *endo*), 3.09 – 3.02 (m, 1H *endo*), 2.94 (br s, 1H *endo*), 2.79 (br s, 1H *endo*+2H *exo*), 2.44 – 2.34 (m, 1H *endo*), 1.86 – 1.79 (m, 1H *endo*), 1.77 – 1.70 (m, 1H *exo*), 1.45 – 1.40 (m, 1H *endo*), 1.35 – 1.22 (m, 1H *endo*+3H *exo*), 1.15 – 1.09 (m, 1H *exo*), 0.52 – 0.46 (m, 1H *endo*). **^{13}C NMR** (126 MHz, $CDCl_3$) δ 139.37, 139.29, 137.33, 137.16, 137.05, 136.79, 136.70, 132.52, 129.23, 129.19, 128.05, 128.02, 75.27, 74.34, 72.68, 72.57, 49.54, 45.16, 44.11, 43.87, 42.32, 41.67, 39.85, 38.95, 33.56, 33.53, 29.86, 29.27. **HRMS** (DART) *m/z* calculated for $C_{16}H_{20}BrO^+$ $[M + H]^+$ 307.06920, found 307.06923.

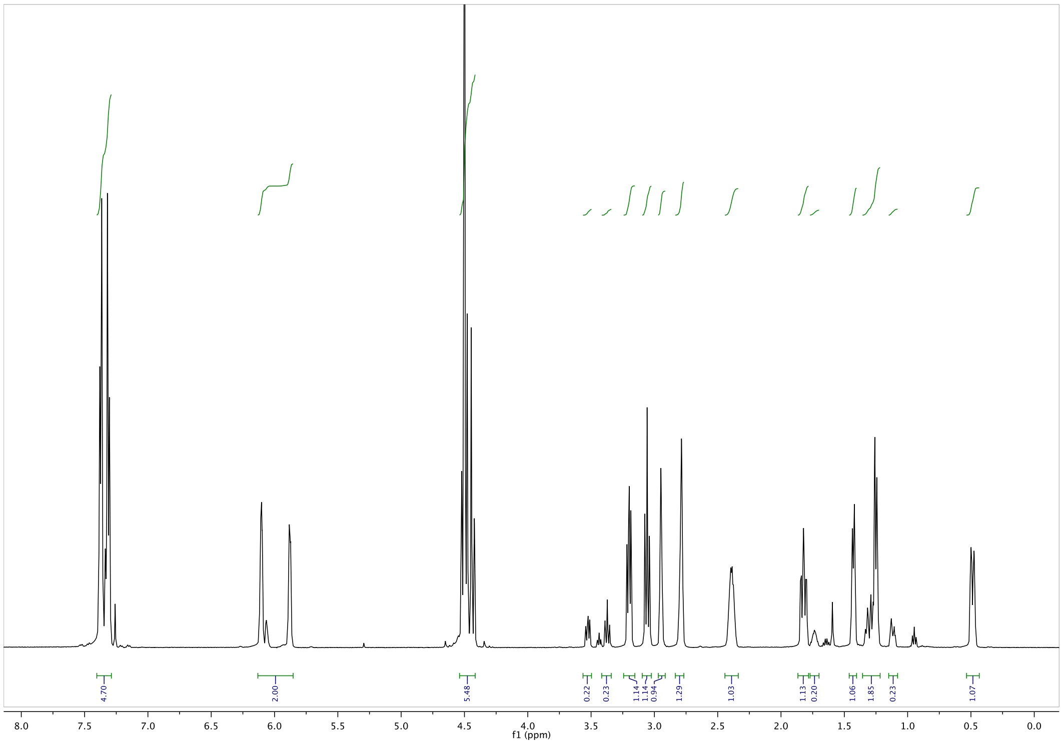

Figure S3. ^{1}H NMR spectrum of **3** in $CDCl_3$.

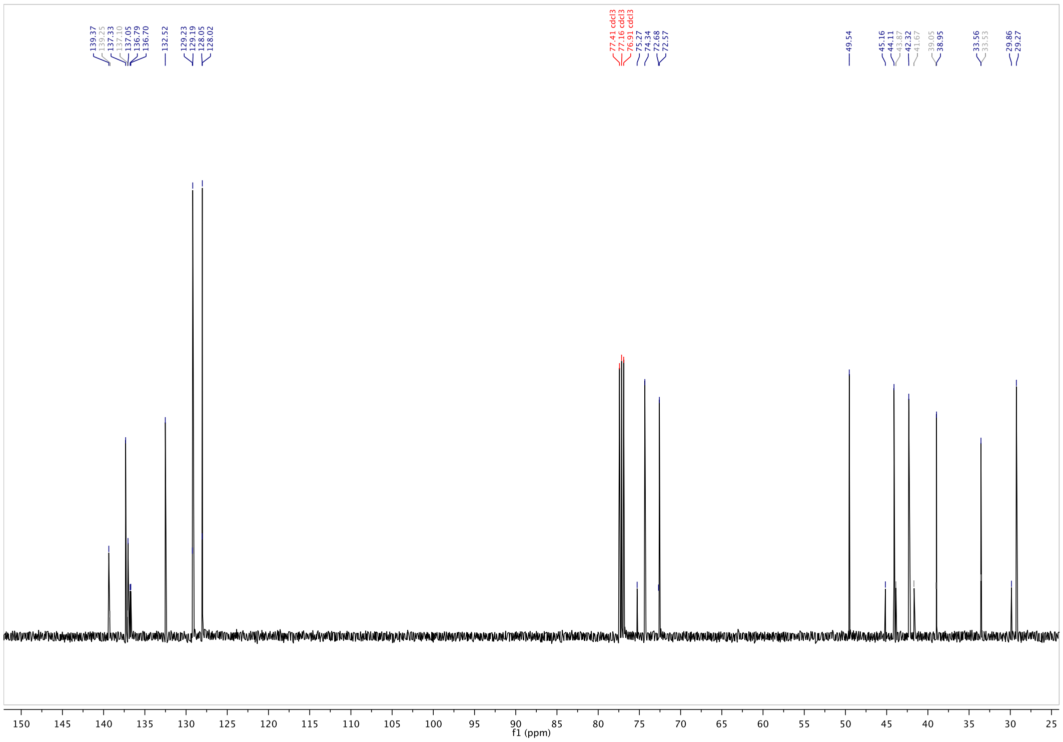

Figure S4. ^{13}C NMR spectrum of **3** in $CDCl_3$.

3.3 Preparation of bis-norbornene terminated PEO crosslinker (1a)

In a N_2 glovebox, a Fischer-Porter bottle was charged with THF (2.0 mL) solution of **2** (108.5 mg, 0.8737 mmol). To this solution was added dropwise 0.31 M THF solution of potassium naphthalenide (2.82 mL, 0.87 mmol) leading to a dark green solution. The bottle was sealed with the reactor head, removed from the glove box and stirred at 22 °C for 1 h. The mixture was cooled with liquid nitrogen and ethylene oxide (1.54 g, 35.0 mmol) was condensed into it. The solution was allowed to warm to room temperature and stirred for 16 h. A THF (0.5 mL) solution of **3** (403 mg, 1.31 mmol) was added under nitrogen to cap the living alkoxide, resulting in immediate precipitation of KBr salt. The mixture was stirred at 50 °C for 6 h, cooled to room temperature and filtered through Celite plug to remove the fine powder salt. The filtrate was concentrated to about 5 mL and added dropwise into hexanes (~200 mL) to precipitate the PEO crosslinker out and remove excess **3**. The white powder was collected by filtration and dried under vacuum overnight (1.70 g, 88%). **^{1}H NMR** (500 MHz, $CDCl_3$) δ 7.31 – 7.28 (m, 4H), 6.12 – 5.82 (m, 4H), 4.56 – 4.38 (m, 4H), 3.63 (s, 202H), 3.38 – 0.43 (m, 18H). **^{13}C NMR** (126 MHz, $CDCl_3$) δ 138.28, 137.59, 137.25, 137.22, 136.75, 136.71, 132.60, 132.55, 127.94, 127.90, 127.78, 127.76, 76.16, 75.19, 75.05, 74.11, 73.15, 72.89, 72.78, 70.69, 70.38, 70.35, 69.48, 49.52, 45.14, 45.11, 44.08, 44.05, 43.85, 43.73, 42.30, 41.64, 39.03, 38.93, 38.88, 38.79, 29.85, 29.82, 29.26. **MS** (MALDI-TOF) *m/z* calculated for $C_{104}H_{190}O_{42}Na^+$ [M (n=40) + Na]$^+$ 2134.26, found 2134.58.

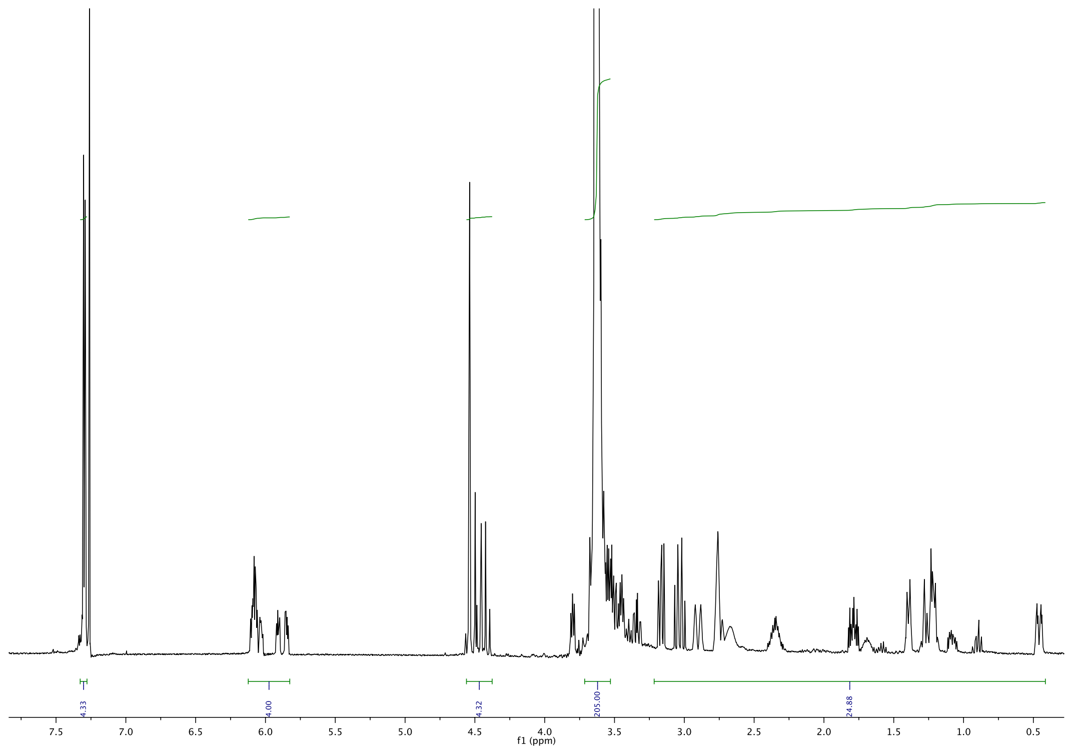

Figure S5. ^{1}H NMR spectrum of **1a** in $CDCl_3$.

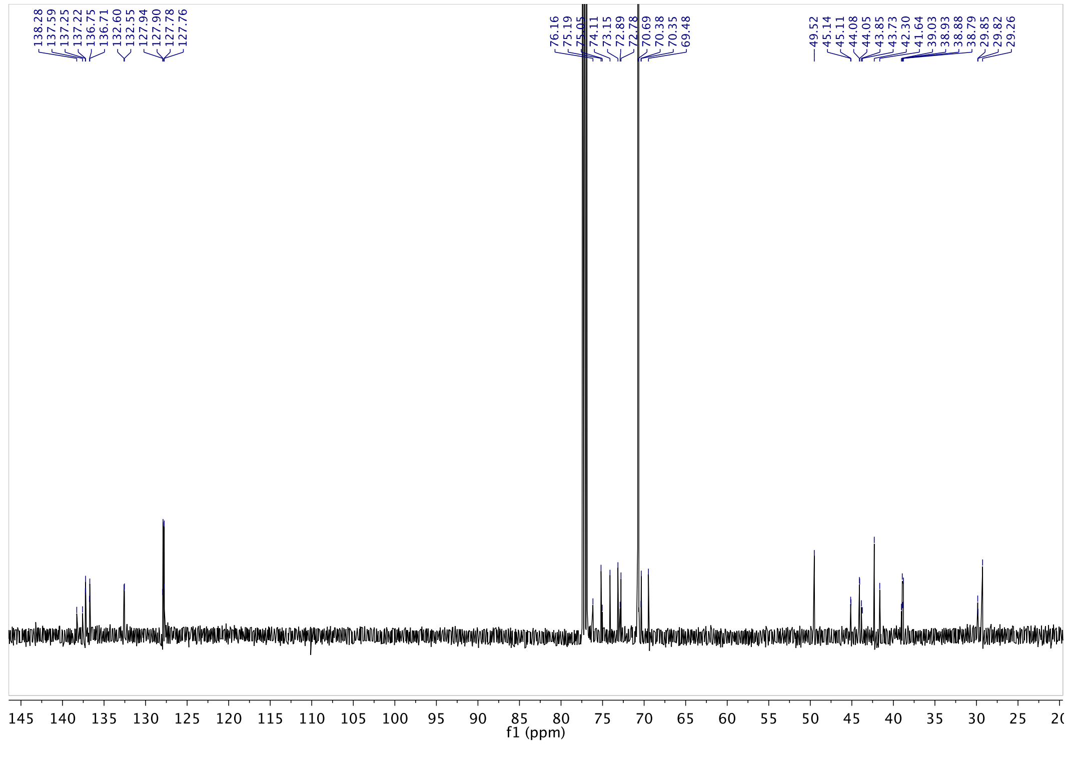

Figure S6. ^{13}C NMR spectrum of **1a** in $CDCl_3$.

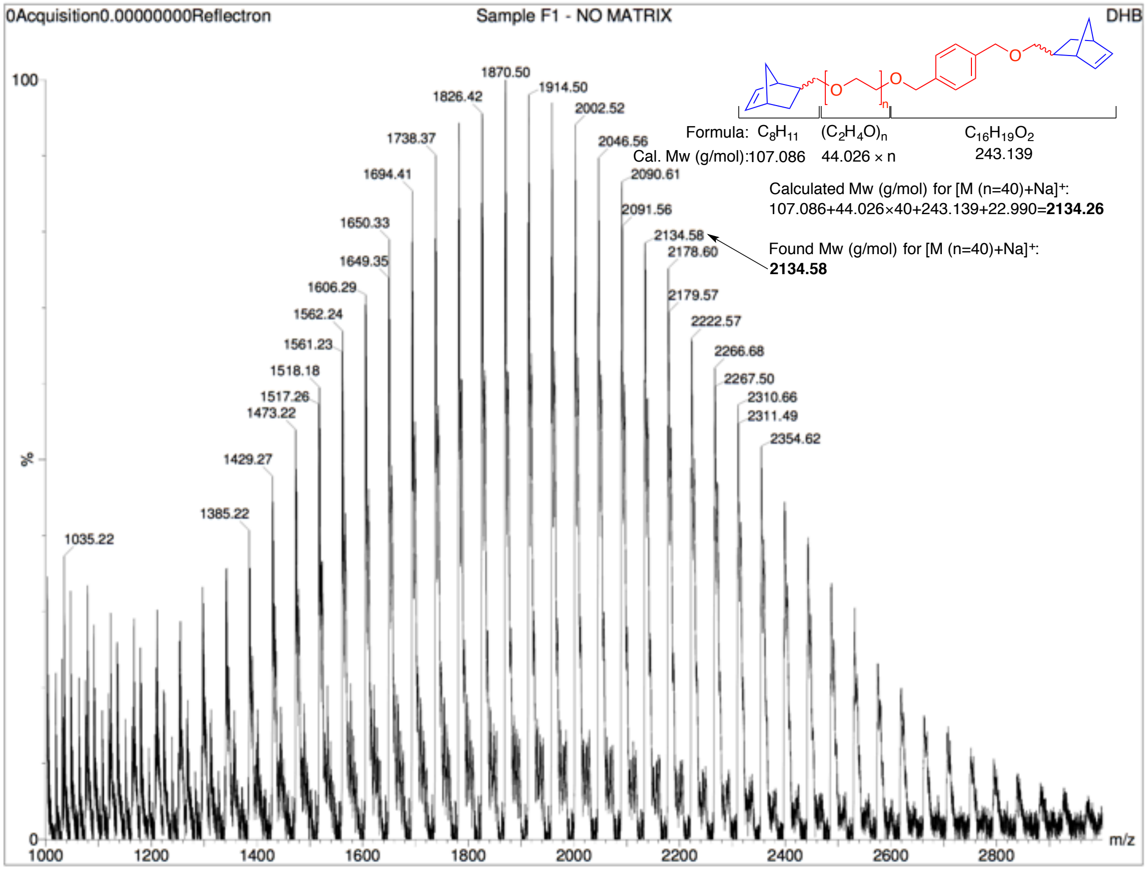

Figure S7. MALDI-TOF mass spectrum of **1a** with dithranol as the matrix.

4. Synthesis of the Cross-Linked Solid Polymer Electrolytes (SPEs)

4.1 Di-Norbornene Terminated PEO Crosslinker

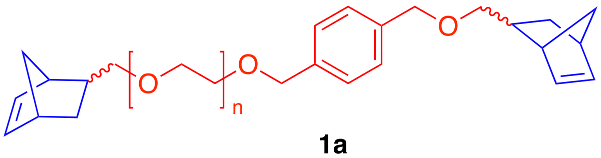

1a

The effect of crosslinker length on ionic conductivity was studied by synthesizing three crosslinkers with different molecular weights using the procedure described above (SI 3.3). The molecular weight and polydispersity index (PDI) are described in Table S1.

Table S1. Di-norbornene terminated PEO crosslinker.

Entry No.	EO units in the crosslinker[a]	M_n–NMR[a] (kg/mol)	M_n–GPC[b] (kg/mol)	PDI[b]
1	52	2.6	2.1	1.3
2	88	4.2	3.2	1.3
3	120	5.5	3.9	1.4

[a]Determined by ^1H NMR spectroscopy. [b]Determined by THF gel permeation chromatography with polystyrene standards at 30 °C.

4.2 Nomenclature of hPNB-PEO, PCOD-PEO Cross-Linked Solid Polymer Electrolyte

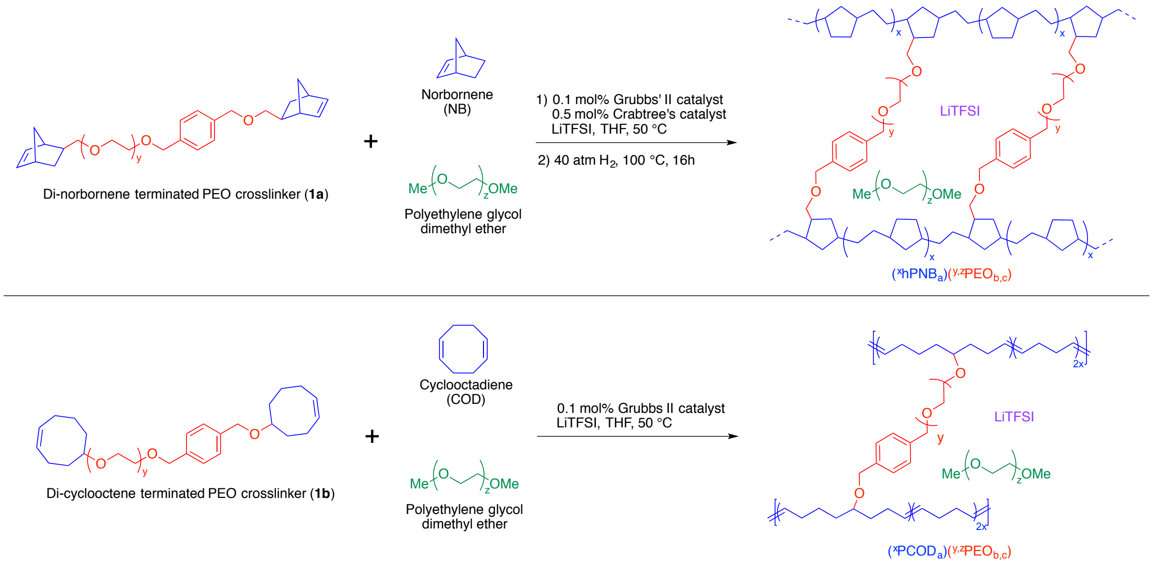

Scheme S2. General scheme for the synthesis of hPNB-PEO, PCOD-PEO cross-linked system.

Nomenclature of hPNB-PEO system

(^{x}hPNB$_a$)(^{y,z}PEO$_{b,c}$)

where

hPNB: hydrogenated polynorbornene; **PEO**: poly(ethylene oxide); **x**: average number of hydrogenated norbornene (hNB) units between two cross-linkers; **y**: average number of ethylene oxide(EO) units in the cross-linker; **z**: average number of EO units in the plasticizer (omitted for unplasticized system); **a**: mole of hNB/ total moles of hNB and EO; **b**: mole of EO from cross-linkers/ total moles of hNB and EO; **c**: mole of EO from

plasticizers/ total moles of hNB and EO.

Calculation:

Define

m = (mmols of norbornene (NB)) + 2 × mmols of **1a**)

x = m / (2 × mmols of **1a**)

y = average number of EO in **1a**

z = 5 (if plasticized)

a = m / (m + y × mmols of **1a** + z × mmols of plasticizer)

b = y × mmols of **1a** / (m + y × mmols of **1a** + z × mmols of plasticizer)

c = z × mmols of plasticizer / (m + y × mmols of **1a** + z × mmols of plasticizer)

Nomenclature of PCOD-PEO system

(^{x}PCOD$_{a}$)(^{y,z}PEO$_{b,c}$)

where

PCOD: polycyclooctadiene; **PEO**: poly(ethylene oxide); **x**: average number of cyclooctadiene (COD) units between two cross-linkers; **y**: average number of ethylene oxide (EO) units in the cross-linker; **z**: average number of EO units in the plasticizer (omitted for unplasticized system); **a**: mole of COD/ total moles of COD and EO; **b**: mole of EO from cross-linkers/ total moles of COD and EO; **c**: mole of EO from plasticizers/ total moles of COD and EO.

Calculation:

Define

m = (mmols of cyclooctadiene (COD)) + 2 × mmols of **1b**)

x = m / (2 × mmols of **1b**)

y = average number of EO in **1b**

z = 5 (if plasticized)

a = m / (m + y × mmols of **1b** + z × mmols of plasticizer)

b = y × mmols of **1b**/ (m + y × mmols of **1b** + z × mmols of plasticizer)

c = z × mmols of plasticizer / (m + y × mmols of **1b** + z × mmols of plasticizer)

4.3 Sample procedure for the synthesis of hPNB-PEO, PCOD-PEO cross-linked system:

The procedure is similar as reported for PE-PEO system. In a N_2 filled glove box, cross-linker **1a** (130.8 mg, 0.03305 mmol) with 90 EO units in the cross-linker was dissolved in 1.0 mL of THF. To this solution was subsequently added norbornene (46.2 mg, 0.491 mmol, in 1 mL THF), LiTFSI (83.3 mg, 0.290 mmol, in 1 mL THF), PEG 275 (123 mg, 0.447 mmol, in 0.5 mL THF, if plasticized), Crabtree's catalyst (2.2 mg, 2.8×10^{-3} mmol, in 0.4 mL $CHCl_3$) and Grubbs' 2nd generation catalyst (0.5 mg, 6×10^{-4} mmol, in 0.5 mL THF). The resulting solution was quickly poured into a cut regular metal pan (Teflon coated, diameter of 5.25 cm and depth of 3.0 cm). The mixture gelled up in less than 1 minute. The teflon-coated metal pan was then covered with the top part of a volume glass chamber and placed on top of the hot plate with a metal plate in between to ensure uniform heating. The film was casted in the glove box at 50 °C for 4 h. After all the solvent evaporated off, ~5 mL hexanes was added to the metal pan to help peeling the film off. The film was dried under vacuum at 22 °C for 24 h and then placed in a Parr reactor and sealed. The Parr reactor was pressurized to 40.8 atm with hydrogen and vented down to 3.4 atm. This process was repeated twice to purge the air out. The Parr reactor was then pressurized to 40.8 atm and heat to 100 °C. After 16 h, the reactor was

cooled and vented. The film was dried under vacuum at 22 °C for 24 h. No solvent peak was seen from the $CDCl_3$ extracting of the film, proving there is no remaining solvent. A residue water content of < 0.01 wt % was determined by Karl-Fisher analysis from a THF extraction of the film. Open-circuit voltage for a Li/SPE/Li symmetric coin cell was measured to be 0.0 V, further proving no remaining solvent and water. A control experiment with films without rigorous drying step showed a 0.4 V open-circuit voltage for the symmetric cell, indicating reactions between Li metal and the residue solvent or water. Films of PCOD-PEO cross-linked systems were cast the same way except Crabtree's catalyst was not added and hydrogenation was not performed.

5. Control Experiment

In order to prove Crabtree's catalyst results in complete hydrogenation of main-chain alkenes, a PEG-grafted NB comonomer was used instead of **1a** during film cast to obtain a soluble hydrogenated copolymer. The synthesis of PEG grafted NB was similar as **1a** except the living alkoxide was quenched by benzyl bromide instead of **3**. In a N_2 glovebox, a Fischer-Porter bottle was charged with THF (2.0 mL) solution of **2** (57.5 mg, 0.463 mmol). To this solution was added dropwise 0.31 M THF solution of potassium naphthalenide (1.49 mL, 0.46 mmol) leading to a dark green solution. The bottle was sealed with the reactor head, removed from the glove box and stirred at 22 °C for 1 h. The mixture was cooled with liquid nitrogen and ethylene oxide (1.63 g, 37.0 mmol) was condensed into it. The solution was allowed to warm to room temperature and stirred for 16 h. Benzyl bromide (0.082 mL, 0.69 mmol) was added under nitrogen to cap the living alkoxide, resulting in immediate precipitation of KBr salt. The mixture was stirred at 50 °C for 6 h, cooled to room temperature and filtered through Celite plug to remove the fine

powder salt. The filtrate was concentrated to about 5 mL and added dropwise into hexanes (~200 mL) to precipitate the PEO crosslinker out. The white powder was collected by filtration and dried under vacuum overnight (1.41 g, 87%). Film cast procedure was similar to hPNB-PEO and PCOD-PEO systems as described in Section **4.3** except PEG-grafted NB was used instead of **1a**. The ^{1}H NMR of hydrogenated film is shown in Figure S8. It can be seen that no peaks were seen at the alkene range (δ 5–6 ppm). All the norbornene units were reduced to saturated hydrocarbons, corresponding to the peaks between δ 0.5 to 2.0 ppm. The peak at δ 3.7 ppm was from PEO. The sharp singlet at δ 4.6 ppm corresponded to the benzylic hydrogens. This result showed that the Crabtree's catalyst gave complete hydrogenation of the polynorbornene backbones.

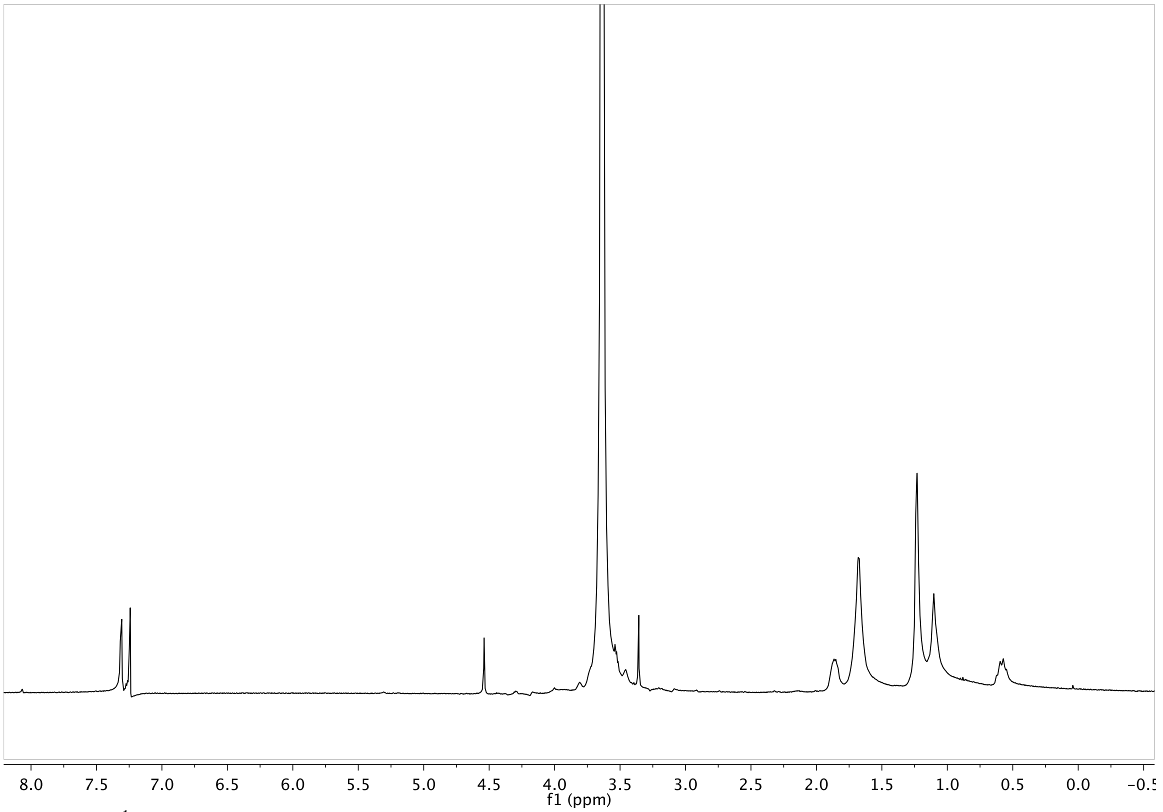

Figure S8. ^{1}H NMR spectrum of hydrogenated film in $CDCl_3$.

6. Differential Scanning Calorimetry (DSC)

DSC analysis was performed using a TA Instruments Q1000 instrument equipped with liquid nitrogen cooling system and automated sampler. Typical DSC samples were made in aluminum pans and the method used was 10 °C/ min ramp, with one cycle of heat, cool, and heat again. The DSC data of plasticized hPNB-PEO and PCOD-PEO systems were shown in the main paper. Unplasticized DSC data were shown in Table S2 and S3.

Table S2. Composition and thermal properties of Unplasticized hPNB-PEO Cross-Linked SPEs[a]

Entry	Unplasticized SPE	[NB]:[1] ratio	hPNB segments[b]			PEO segments[c]		
			T_m[d] (°C)	ΔH_{fus}[d] (J/g)	T_g[d] (°C)	T_c[d] (°C)	T_m[d] (°C)	ΔH_{fus}[d] (J/g)
1	$(^{8.5}hPN_{0.25})(^{52}PEO_{0.75})$	15:1	97	8.8	−46	*n.d.*[e]	*n.d.*[e]	*n.d.*[e]
2	$(^{6}hPN_{0.19})(^{52}PEO_{0.81})$	10:1	93	3.8	−45	*n.d.*[e]	*n.d.*[e]	*n.d.*[e]
3	$(^{4.5}hPN_{0.15})(^{52}PEO_{0.85})$	7:1	*n.d.*[e]	*n.d.*[e]	−47	*n.d.*[e]	23	1.1
4	$(^{8.5}hPN_{0.16})(^{88}PEO_{0.84})$	15:1	92	2.8	−48	−6	32	27.9
5	$(^{6}hPN_{0.12})(^{88}PEO_{0.88})$	10:1	*n.d.*[e]	*n.d.*[e]	−49	−5	32	31.3
6	$(^{4.5}hPN_{0.09})(^{88}PEO_{0.91})$	7:1	*n.d.*[e]	*n.d.*[e]	−50	−6	32	38.5
7	$(^{8.5}hPN_{0.12})(^{140}PEO_{0.89})$	15:1	*n.d.*[e]	*n.d.*[e]	−36	−7	36	49.0
8	$(^{6}hPN_{0.09})(^{140}PEO_{0.92})$	10:1	*n.d.*[e]	*n.d.*[e]	−39	−6	36	49.8
9	$(^{4.5}hPN_{0.07})(^{140}PEO_{0.94})$	7:1	*n.d.*[e]	*n.d.*[e]	−39	−9	37	60.7

[a]All films have [EO]:[Li] composition of 20:1, where EO means ethylene oxide units in the crosslinker. [b]hPNB segments: hydrogenated polynorbornene domains in the polymer electrolytes. [c]PEO segments: polyethylene oxide domains in the polymer electrolytes. [d]Glass transition temperature (T_g), cold crystallization temperature (T_c), melting temperature (T_m) and enthalpy of fusion (ΔH_{fus}) were determined by differential scanning calorimetry from the second heat cycle. [e]Not detected.

Table S3. Composition and thermal properties of Unplasticized PCOD-PEO Cross-Linked SPEs[a]

Entry	Unplasticized SPE	[COD]:[2] ratio	PEO segments[b]		
			T_g[c] (°C)	T_m[c] (°C)	ΔH_{fus}[c] (J/g)
1	$(^{8.5}PCOD_{0.30})(^{40}PEO_{0.70})$	15:1	−21	*n.d.*[d]	*n.d.*[d]
2	$(^{6}PCOD_{0.23})(^{40}PEO_{0.77})$	10:1	−30	*n.d.*[d]	*n.d.*[d]
3	$(^{4.5}PCOD_{0.28})(^{40}PEO_{0.82})$	7:1	−34	*n.d.*[d]	*n.d.*[d]
4	$(^{8.5}PCOD_{0.18})(^{75}PEO_{0.82})$	15:1	−41	*n.d.*[d]	*n.d.*[d]
5	$(^{6}PCOD_{0.14})(^{75}PEO_{0.86})$	10:1	−40	*n.d.*[d]	*n.d.*[d]
6	$(^{4.5}PCOD_{0.11})(^{75}PEO_{0.89})$	7:1	−42	*n.d.*[d]	*n.d.*[d]
7	$(^{8.5}PCOD_{0.10})(^{160}PEO_{0.90})$	15:1	−39	39	68.1
8	$(^{6}PCOD_{0.07})(^{160}PEO_{0.93})$	10:1	−39	39	61.5
9	$(^{4.5}PCOD_{0.05})(^{160}PEO_{0.95})$	7:1	−40	39	76.1

[a]All films have [EO]:[Li] composition of 20:1, where EO means ethylene oxide units in the crosslinker. [b]PEO segments: polyethylene oxide domains in the polymer electrolytes. [c]Glass transition temperature (T_g), cold crystallization temperature (T_c), melting temperature (T_m) and enthalpy of fusion (ΔH_{fus}) were determined by differential scanning calorimetry from the second heat cycle. [d]Not detected.

7. DC Ionic conductivity and VTF fitting

Samples for DC ionic conductivity were prepared in a dry glove box. The

polymer electrolyte membrane was cut into a circle with a diameter of 1 cm using hole punch. The thickness of the electrolyte was typically in the range of 150 – 200 μm. The cut membrane was then sandwiched between two gold plated stainless steel electrodes to form a symmetric cell. The DC ionic conductivity was determined from the plateau value of the conductivity as a function of frequency as reported for PE-PEO system. Figure S9 shows one example of the plots of the real part of the ionic conductivity and tan (delta) vs. frequency. The conductivity was recorded at the frequency when tan (delta) reached the maximum. Each sample was measured three times at each temperature and took an average. DC ionic conductivity data for unplasticized and plasticized hPNB-PEO and PCOD-PEO systems were reported in Table S4, S5, S6, S7. VTF fitting in the main text was done using the variable temperature DC ionic conductivity.

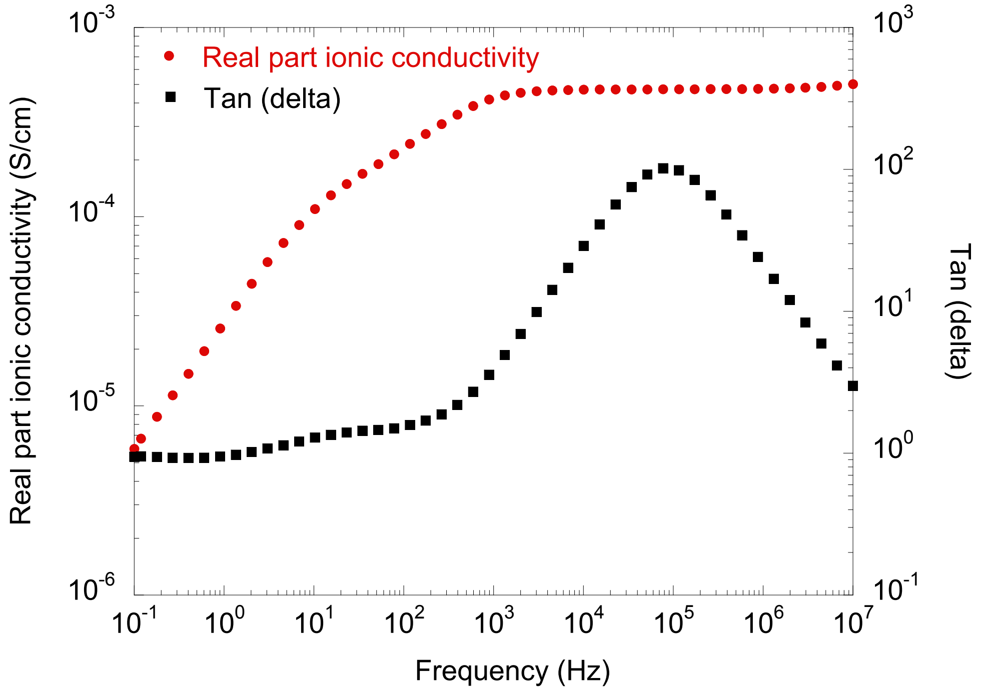

Figure S9. Real part of the ionic conductivity and tan (delta) vs. frequency plot for $(^{8.5}\text{hPNB}_{0.11})(^{88,5}\text{PEO}_{0.46,0.43})$.

Table S4. DC ionic Conductivities of Unplasticized hPNB-PEO Cross-Linked SPEs[a]

Entry	Unplasticized SPE	DC Ionic Conductivity (S/cm)						
		10 °C	25 °C	40 °C	55 °C	70 °C	85 °C	100 °C
1	$(^{8.5}hPN_{0.25})(^{52}PEO_{0.75})$	2.4×10^{-6}	1.1×10^{-5}	3.6×10^{-5}	8.8×10^{-5}	1.8×10^{-4}	3.3×10^{-4}	5.2×10^{-4}
2	$(^{6}hPN_{0.19})(^{52}PEO_{0.81})$	2.3×10^{-6}	1.2×10^{-5}	4.4×10^{-5}	1.2×10^{-4}	2.4×10^{-4}	4.6×10^{-4}	7.4×10^{-4}
3	$(^{4.5}hPN_{0.15})(^{52}PEO_{0.85})$	3.6×10^{-6}	1.7×10^{-5}	5.6×10^{-5}	1.4×10^{-4}	2.8×10^{-4}	4.9×10^{-4}	7.8×10^{-4}
4	$(^{8.5}hPN_{0.16})(^{88}PEO_{0.84})$	6.3×10^{-6}	2.8×10^{-5}	8.5×10^{-5}	2.0×10^{-4}	3.8×10^{-4}	6.6×10^{-4}	1.0×10^{-3}
5	$(^{6}hPN_{0.12})(^{88}PEO_{0.88})$	6.3×10^{-6}	3.0×10^{-5}	1.2×10^{-4}	2.6×10^{-4}	5.0×10^{-4}	8.5×10^{-4}	1.3×10^{-3}
6	$(^{4.5}hPN_{0.09})(^{88}PEO_{0.91})$	3.7×10^{-6}	2.3×10^{-5}	1.1×10^{-4}	2.7×10^{-4}	5.3×10^{-4}	9.1×10^{-4}	1.4×10^{-3}
7	$(^{8.5}hPN_{0.12})(^{140}PEO_{0.89})$	9.3×10^{-7}	6.8×10^{-6}	7.7×10^{-5}	2.4×10^{-4}	4.5×10^{-4}	7.5×10^{-4}	1.1×10^{-3}
8	$(^{6}hPN_{0.09})(^{140}PEO_{0.92})$	2.3×10^{-6}	1.5×10^{-5}	1.0×10^{-4}	3.3×10^{-4}	6.3×10^{-4}	1.1×10^{-3}	1.6×10^{-3}
9	$(^{4.5}hPN_{0.07})(^{140}PEO_{0.94})$	2.4×10^{-6}	1.5×10^{-5}	1.0×10^{-4}	3.6×10^{-4}	6.8×10^{-4}	1.1×10^{-3}	1.8×10^{-3}

[a]All films had [EO]:[Li] composition of 20:1; where EO means ethylene oxide units in the cross-linker. Conductivities were determined by dielectric spectroscopy measurements.

Table S5. DC ionic Conductivities of Unplasticized PCOD-PEO Cross-Linked SPEs[a]

Entry	Unplasticized SPE	DC Ionic Conductivity (S/cm)						
		10 °C	25 °C	40 °C	55 °C	70 °C	85 °C	100 °C
1	$(^{8.5}PCOD_{0.30})(^{40}PEO_{0.70})$	3.5×10^{-6}	1.4×10^{-5}	3.8×10^{-5}	8.7×10^{-5}	1.6×10^{-4}	2.7×10^{-4}	4.2×10^{-4}
2	$(^{6}PCOD_{0.23})(^{40}PEO_{0.77})$	5.2×10^{-6}	2.2×10^{-5}	6.3×10^{-5}	1.5×10^{-4}	2.8×10^{-4}	4.6×10^{-4}	7.0×10^{-4}
3	$(^{4.5}PCOD_{0.28})(^{40}PEO_{0.82})$	6.5×10^{-6}	2.8×10^{-5}	8.1×10^{-5}	1.9×10^{-4}	3.5×10^{-4}	5.8×10^{-4}	8.6×10^{-4}
4	$(^{8.5}PCOD_{0.18})(^{75}PEO_{0.82})$	7.2×10^{-6}	2.9×10^{-5}	8.4×10^{-5}	1.9×10^{-4}	3.5×10^{-4}	5.8×10^{-4}	8.6×10^{-4}
5	$(^{6}PCOD_{0.14})(^{75}PEO_{0.86})$	1.1×10^{-5}	4.0×10^{-5}	1.1×10^{-4}	2.3×10^{-4}	4.2×10^{-4}	6.7×10^{-4}	9.8×10^{-4}
6	$(^{4.5}PCOD_{0.11})(^{75}PEO_{0.89})$	1.6×10^{-5}	6.7×10^{-5}	1.9×10^{-4}	4.4×10^{-4}	8.1×10^{-4}	1.3×10^{-3}	1.9×10^{-3}
7	$(^{8.5}PCOD_{0.10})(^{160}PEO_{0.90})$	5.4×10^{-7}	4.4×10^{-6}	4.8×10^{-5}	3.4×10^{-4}	6.3×10^{-4}	1.0×10^{-3}	1.6×10^{-3}
8	$(^{6}PCOD_{0.07})(^{160}PEO_{0.93})$	2.6×10^{-6}	1.7×10^{-5}	1.5×10^{-4}	7.2×10^{-4}	1.3×10^{-3}	2.2×10^{-3}	3.2×10^{-3}
9	$(^{4.5}PCOD_{0.05})(^{160}PEO_{0.95})$	2.0×10^{-6}	1.5×10^{-5}	1.7×10^{-4}	7.4×10^{-4}	1.4×10^{-3}	2.2×10^{-3}	3.2×10^{-3}

[a]All films had [EO]:[Li] composition of 20:1; where EO means ethylene oxide units in the cross-linker. Conductivities were determined by dielectric spectroscopy measurements.

Table S6. DC ionic Conductivities of Plasticized hPNB-PEO Cross-Linked SPEs[a]

Entry	Plasticized SPE	DC Ionic Conductivity (S/cm)						
		10 °C	25 °C	40 °C	55 °C	70 °C	85 °C	100 °C
1	$(^{8.5}hPNB_{0.25})(^{88,0}PEO_{0.75,0})$	6.3×10^{-6}	2.8×10^{-5}	8.5×10^{-5}	2.0×10^{-4}	3.8×10^{-4}	6.6×10^{-4}	1.0×10^{-3}
2	$(^{8.5}hPNB_{0.14})(^{88,5}PEO_{0.64,0.22})$	2.8×10^{-5}	1.0×10^{-4}	2.5×10^{-4}	4.9×10^{-4}	8.4×10^{-4}	1.3×10^{-3}	1.8×10^{-3}
3	$(^{8.5}hPNB_{0.13})(^{88,5}PEO_{0.57,0.30})$	6.1×10^{-5}	1.9×10^{-4}	4.4×10^{-4}	8.4×10^{-4}	1.4×10^{-3}	2.2×10^{-3}	3.0×10^{-3}
4	$(^{8.5}hPNB_{0.11})(^{88,5}PEO_{0.46,0.43})$	1.8×10^{-4}	4.7×10^{-4}	9.9×10^{-4}	1.7×10^{-3}	2.7×10^{-3}	3.9×10^{-3}	5.3×10^{-3}
5	$(^{8.5}hPNB_{0.09})(^{88,5}PEO_{0.38,0.54})$	3.2×10^{-4}	8.1×10^{-4}	1.7×10^{-3}	2.9×10^{-3}	4.4×10^{-3}	6.3×10^{-3}	8.4×10^{-3}

[a]All films had [EO]:[Li] composition of 20:1; where EO means ethylene oxide units in the cross-linker and plasticizers. Conductivities were determined by dielectric spectroscopy measurements.

Table S7. DC ionic Conductivities of Plasticized PCOD-PEO Cross-Linked SPEs[a]

Entry	Plasticized SPE	DC Ionic Conductivity (S/cm)						
		10 °C	25 °C	40 °C	55 °C	70 °C	85 °C	100 °C
1	$(^{8.5}PCOD_{0.17})(^{75,0}PEO_{0.83,0})$	7.2×10^{-6}	2.9×10^{-5}	8.4×10^{-5}	1.9×10^{-4}	3.5×10^{-4}	5.8×10^{-4}	8.6×10^{-4}
2	$(^{8.5}PCOD_{0.13})(^{75,5}PEO_{0.66,0.21})$	2.4×10^{-5}	8.0×10^{-5}	2.0×10^{-4}	4.1×10^{-4}	7.2×10^{-4}	1.1×10^{-3}	1.6×10^{-3}
3	$(^{8.5}PCOD_{0.11})(^{75,5}PEO_{0.57,0.32})$	4.6×10^{-5}	1.5×10^{-4}	3.7×10^{-4}	7.3×10^{-4}	1.2×10^{-3}	1.9×10^{-3}	2.6×10^{-3}
4	$(^{8.5}PCOD_{0.10})(^{75,5}PEO_{0.48,0.43})$	8.8×10^{-5}	2.6×10^{-4}	6.0×10^{-4}	1.1×10^{-3}	1.9×10^{-3}	2.7×10^{-3}	3.8×10^{-3}
5	$(^{8.5}PCOD_{0.08})(^{75,5}PEO_{0.39,0.53})$	1.1×10^{-4}	3.3×10^{-4}	7.5×10^{-4}	1.4×10^{-3}	2.3×10^{-3}	3.4×10^{-3}	4.6×10^{-3}

[a]All films had [EO]:[Li] composition of 20:1; where EO means ethylene oxide units in the cross-linker and plasticizers. Conductivities were determined by dielectric spectroscopy measurements.

8. Galvanostatic Cycling Measurements

Li/SPE/Li symmetric coin cells were assembled in an argon filled MBraun glovebox, and the galvanostatic cycling short-circuit measurements were performed using a Neware CT-3008 battery tester with wiring into (Fisher Scientific and VWR) convection ovens at a temperature of 90 ^{o}C. An initial 24 hour period of three hour charge and three hour discharge cycling at a lower current density (0.026 mA/cm^2) was performed, followed by each of half cycle of 3 h at 0.26 mA/cm^2 until a sudden drop in voltage was observed. Figure S10 shows the voltage profile of hPNB-PEO as an example.

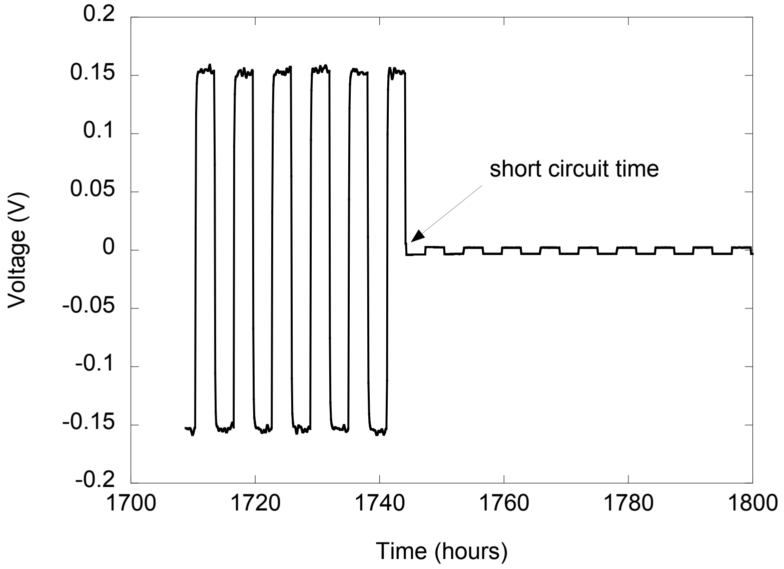

Figure S10. Galvanostatic cycling curve obtained for hPNB-PEO with 32 wt % plasticizer at fixed current density for 0.26 mA/cm^2 and 90 °C. The short circuit time (t_{SC}) is pointed out; C_d value is 1638 C/cm^2.

9. Galvanostatic Polarization Measurements

Galvanostatic polarization measurements were performed on Li/SPE/Li symmetric coin cells by a Neware CT-3008 battery tester. A typical polarization curve at current density of 0.26 mA/cm^2 at 90 °C is shown in Figure S11.

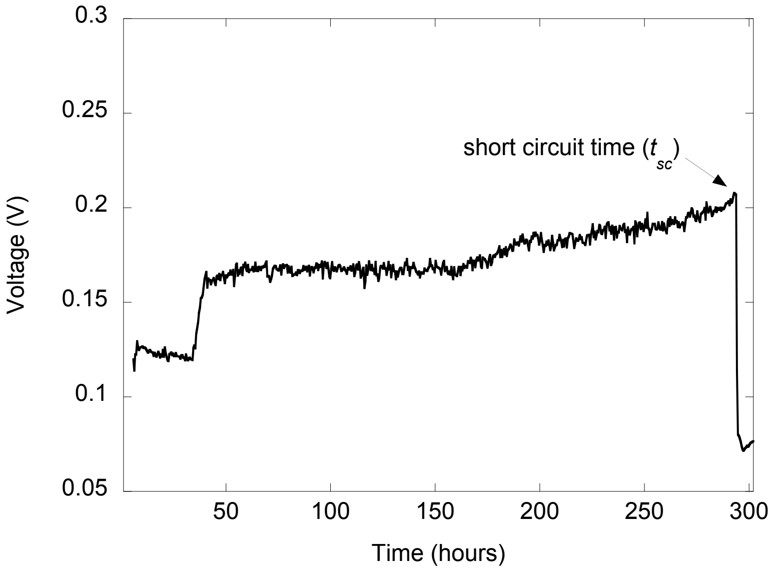

Figure S11. Voltage profile of galvanostatic polarization of hPNB-PEO system at fixed current density of 0.26 mA/cm^2 at 90 °C. The short circuit time is determined at the moment when the sudden voltage drop is observed.

10. Rheology

Anton Paar Physica MCR 301 rheometer was used for rheological measurements with 10 mm diameter parallel plates. The storage G' and loss G'' moduli were quantified as a function of applied angular frequency at low strain (0.1%) and 90 °C.

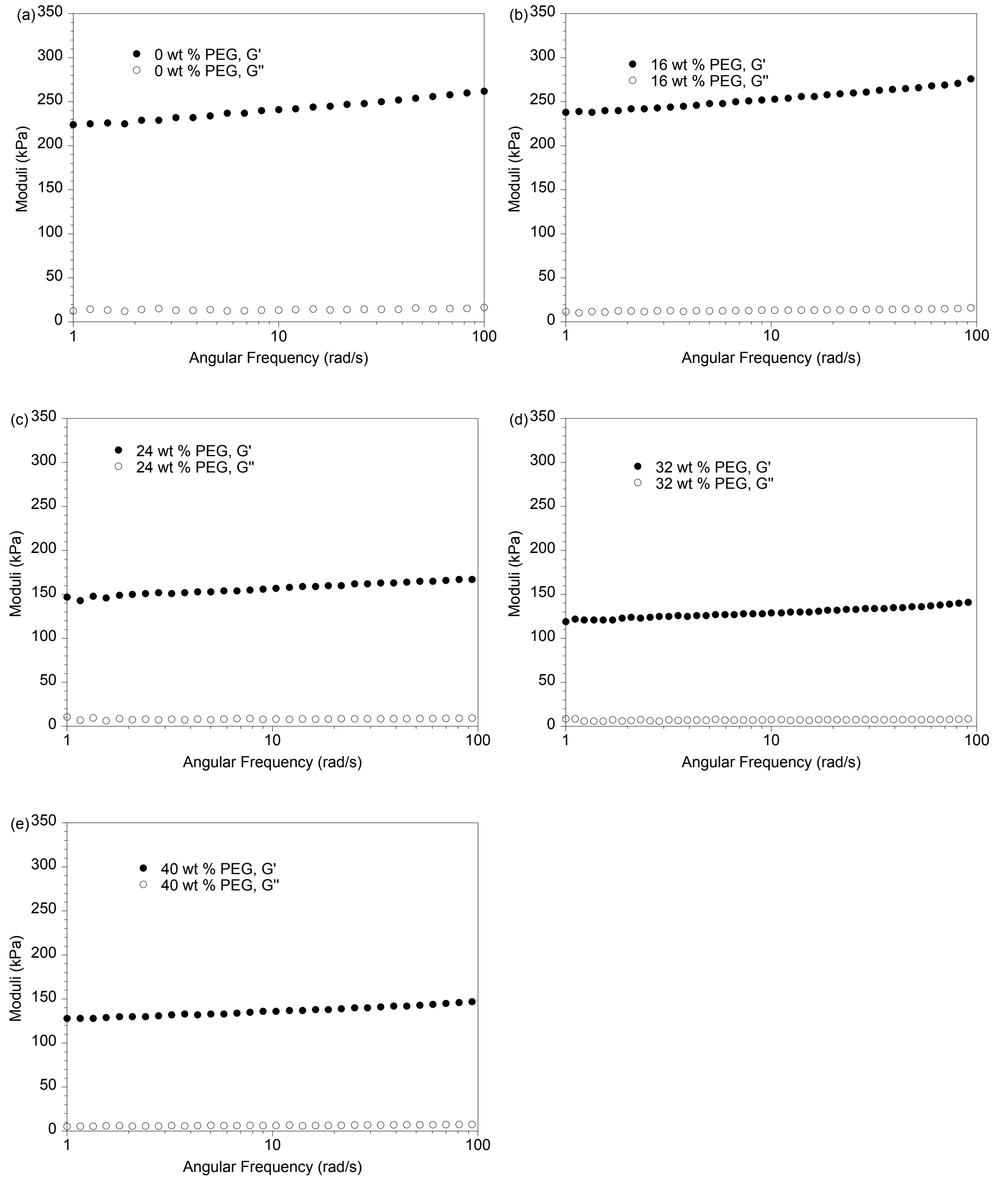

Figure S12. Rheological measurements on hPNB-PEO system with different weight% of the plasticizer at 90 °C. All films had [NB]:[**1a**] ratio of 15:1 and [EO]:[Li] composition of 20:1. Storage modulus $G'(\omega)$ is shown with filled symbols, and the loss modulus $G''(\omega)$ is shown with hollow symbols. (a) 0 wt %, (b) 16 wt%, (c) 24 wt %, (d) 32 wt %, and (e) 40 w % PEG275 plasticizer in the cross-linked films.

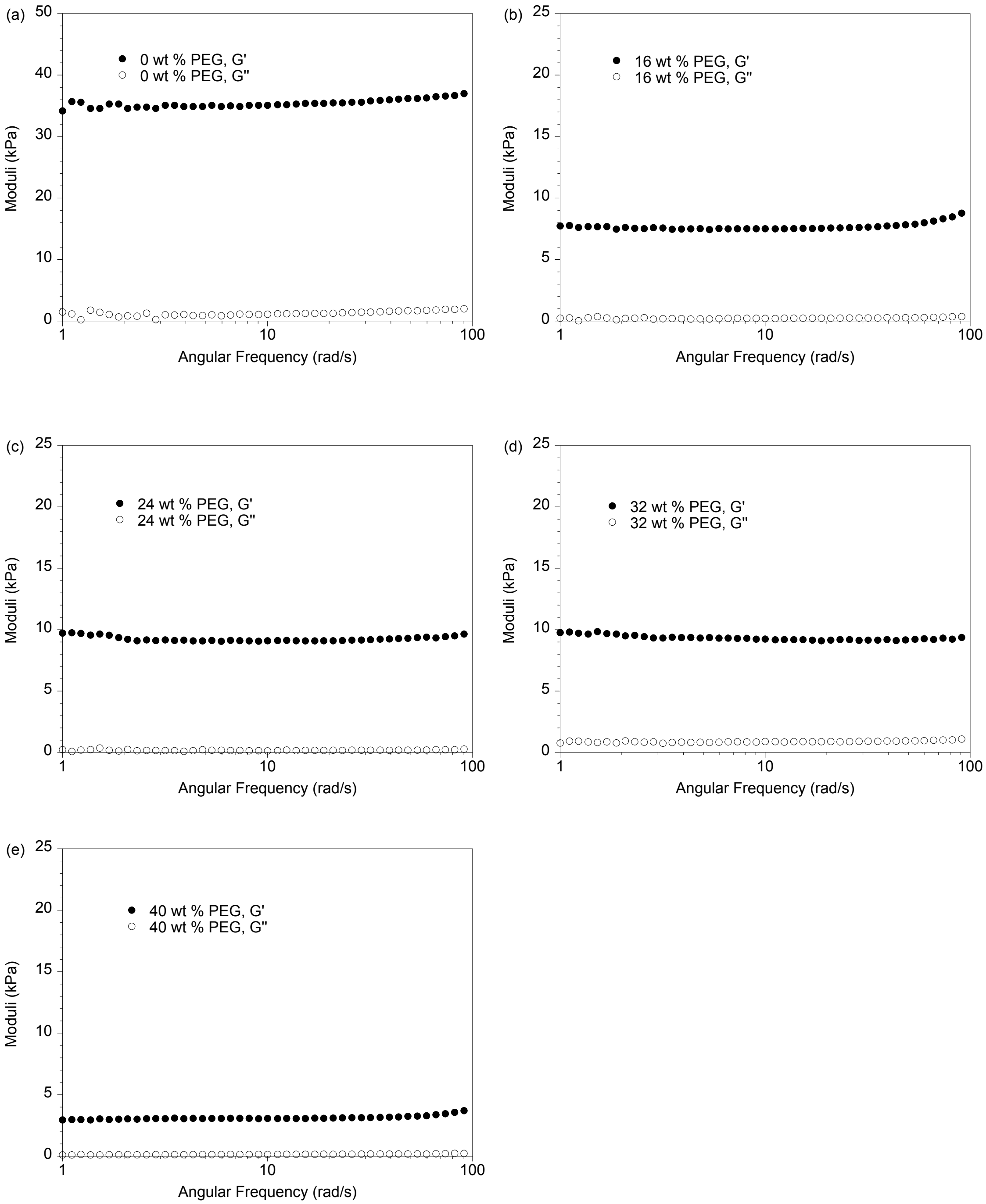

Figure S13. Rheological measurements on PCOD-PEO system with different weight% of the plasticizer at 90 °C. All films had [COD]:[**1b**] ratio of 15:1 and [EO]:[Li] composition of 20:1. Storage modulus $G'(\omega)$ is shown with filled symbols, and the loss modulus $G''(\omega)$ is shown with hollow symbols. (a) 0 wt %, (b) 16 wt%, (c) 24 wt %, (d) 32 wt %, and (e) 40 wt % PEG275 plasticizer in the cross-linked films.

11. SEM of the lithium metal after short-circuit

The morphology of the lithium metal in different systems after short-circuit is analyzed using Keck scanning electron microscope (SEM) at 3kV acceleration voltage. The batteries after short-circuit were disassembled in the glove box and the lithium metal was taken out for observation.

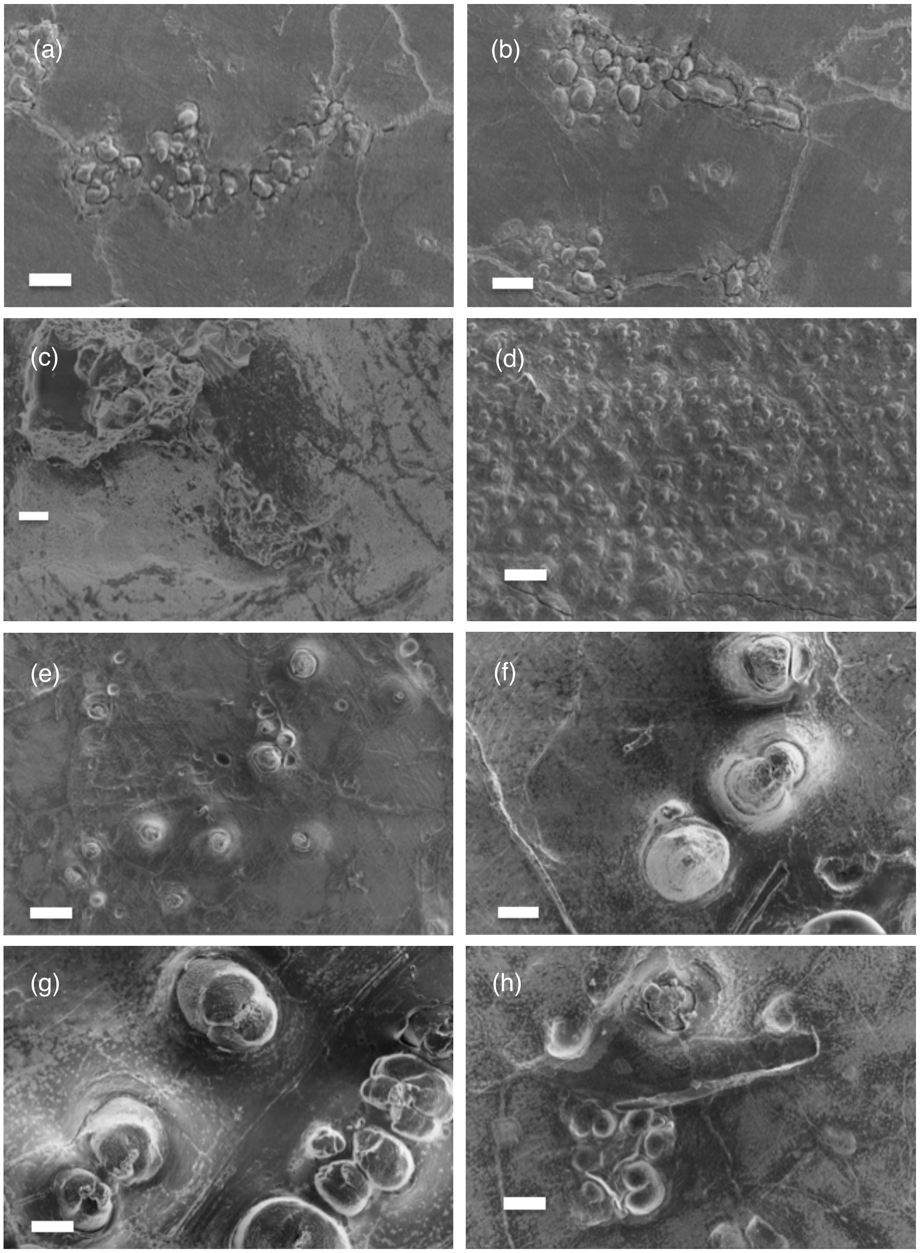

Figure S14. SEM images of lithium metal after short-circuit. Galvanostatic polarization of hPNB-PEO system at (a) 0.26 mA/cm^2; (b) 0.4 mA/cm^2; (c) 1 mA/cm^2; (d) Galvanostatic cycling of hPNB-PEO system

at 0.26 mA/cm^2; Galvanostatic polarization of PCOD-PEO system at (e) 0.26 mA/cm^2; (f) 0.4 mA/cm^2; (g) 1 mA/cm^2; (h) Galvanostatic cycling of PCOD-PEO system at 0.26 mA/cm^2. Scale bar = 20 µm.

11. Electrochemical Impedance Spectroscopy (EIS)

The ac impedance spectroscopy measurements for the Li/SPE/Li symmetric coin cells were measured using a Novocontrol Broadband Dielectric Spectrometer, fitted with a Quatro temperature control system at frequency ranging from 2 KHz to 900 MHz and at amplitude of 10 mV. Figure S15 (a) shows the impedance spectra for hPNB-PEO electrolytes with varied plasticizer weight at 18 °C. With the increase in the amount of PEG275 as plasticizer in the SPEs, the bulk resistance (R_b) of the polymer electrolytes decreases significantly, which is consistent with the expectation. Also with the increase of temperature from 18 °C to 90 °C, both the bulk resistance (R_b) and interfacial resistance (R_i) shows obvious decrease. Figure S15 (c) and (d) shows the corresponding impedance data of the PCOD-PEO system. Consistent with the trend of the conductivity measurement of the different systems (Figure S15), PCOD-PEO shows lower bulk resistance compared to hPNB-PEO system.

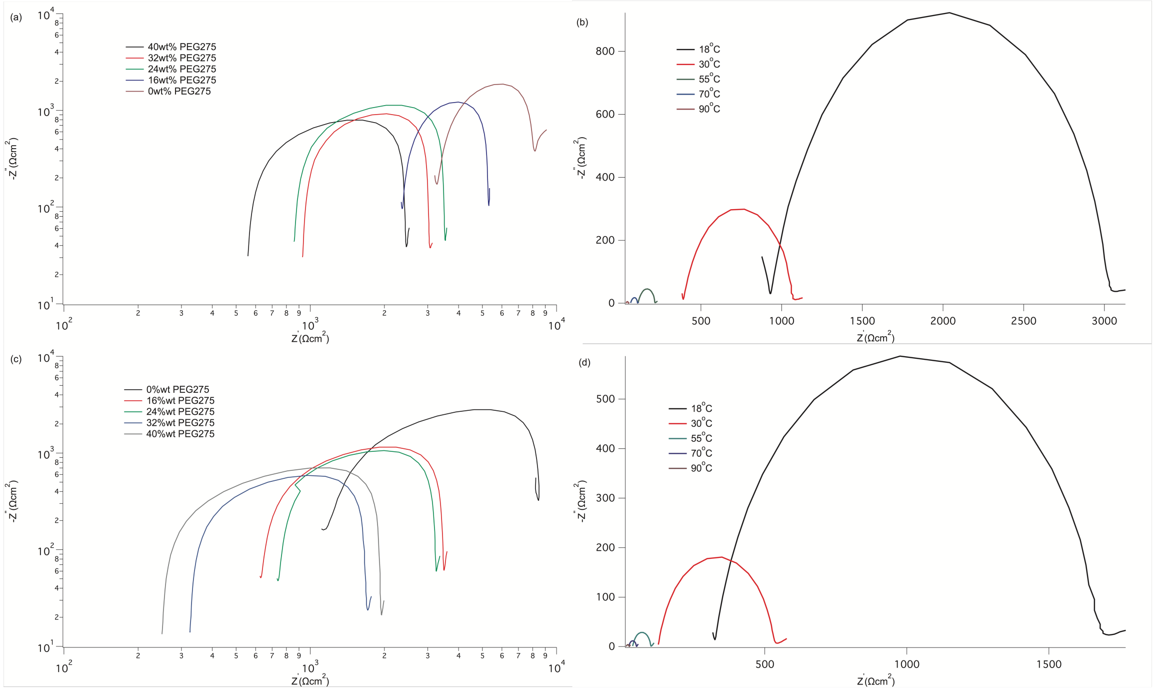

Figure S15. (a) Impedance spectra for hPNB-PEO electrolytes with varied plasticizer weight at 18 °C. (b) Impedance for hPNB-PEO with 32 wt % plasticizer at variable temperature. (c) Impedance spectra for PCOD-PEO electrolytes with varied plasticizer weight at 18 °C. (d) Impedance for PCOD-PEO with 32 wt % plasticizer at variable temperature.

12. Density measurements

The densities for nonplasticized and plasticized hPNB and PCOD electrolytes were measured and summarized in Table S8. The polymer electrolytes were cut into a circle with a hole punch. The thickness was measured using a micrometer. The volume of the membrane can then be calculated by $V = \pi r^2 h$, where V is the volume, r is the diameter of the circle and h is the thickness of the membrane. The masses of the membranes were weighted. The density can then be calculated by $\rho = m/V$, where ρ is the density and m is the mass.

Table S8. Density of the hPNB and PCOD SPEs

hPNB (wt % of PEG250)	Density (g/cm^3)	PCOD (wt % of PEG250)	Density (g/cm^3)
0	1.27	0	1.19
16	1.25	16	1.22
24	1.25	24	1.24
32	1.28	32	1.25
40	1.20	40	1.21

The densities of the both copolymers are around 1.2 – 1.3 g/cm^3 and no significant trend is seen by varying the amount of plasticizers. This result is expected because the densities of the hydrocarbon polymers are all in the range between 0.9 – 1.0 g/ml and are very close to each other (PE ~ 0.97 g/cm^3, hPNB ~ 0.99 g/cm^3, PCOD ~ 0.91 g/cm^3). PEO has a density of about 1.2 g/cm^3 and LiTFSI has a density of about 1.33 g/cm^3. A density around 1.2 g/cm^3 is a reasonable value. Because the densities are very close to each other in both hPNB and PCOD electrolytes, we conclude that the density should have no significant effect on dendrite suppression.

13. Li transference number

The lithium transference number of hPNB-PEO and PCOD-PEO was measured using a conventional Bruce and Vincent method.[3] Specifically, a lithium symmetric cell was assembled with two lithium metal pieces as electrodes and the SPE as the separator. The transference number was then determined using the equation:

$$T_+ = \frac{I^s(\Delta V - I^o R_1^o)}{I^o(\Delta V - I^s R_1^s)}$$

Here ΔV is the potential applied across the cell; R_1^o and R_1^s are the initial and steady state resistances of the passivating layers; I^o and I^s are the initial and steady-state currents respectively. A small ΔV = 20 mV was applied and the result is summarized in Figure S16, which shows the impedance and current change before and after polarization of the cell with unplasticized hPNB-PEO as the separator. The Li transference number is then

calculated as 0.30. A transference number of 0.32 is calculated with unplasticized PCOD-PEO as the separator using the same method.

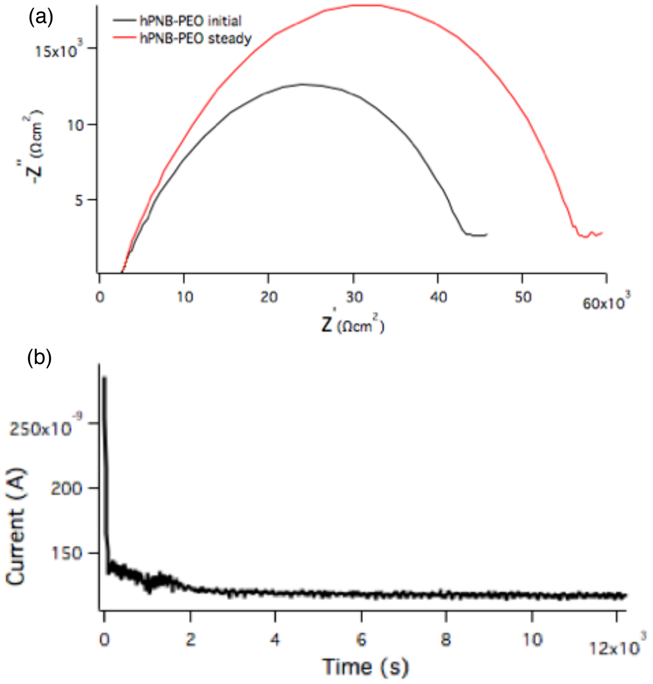

Figure S16. (a) Initial and steady state impedance spectra of lithium symmetric cell for unplasticized hPNB-PEO. (b) Steady state current measurement for unplasticized hPNB-PEO.

14. Battery device test

We applied the 32 wt% plasticized hPNB-PEO membrane to a half cell and the voltage profile is shown below (Figure S17). The half cell battery is composed of $LiCoO_2$ as the cathode, Li metal as the anode and hPNB as the separator. The battery was cycled at 0.5 C. It shows that the battery device still has the feature of $LiCoO_2$ electrode, with a discharge plateau at ~3.9 V, indicating the potential application of the polymer electrolytes in practical battery devices. The capacity is lower than the theoretical capacity of $LiCoO_2$, which is probably due to the limited conductivity of the membrane.

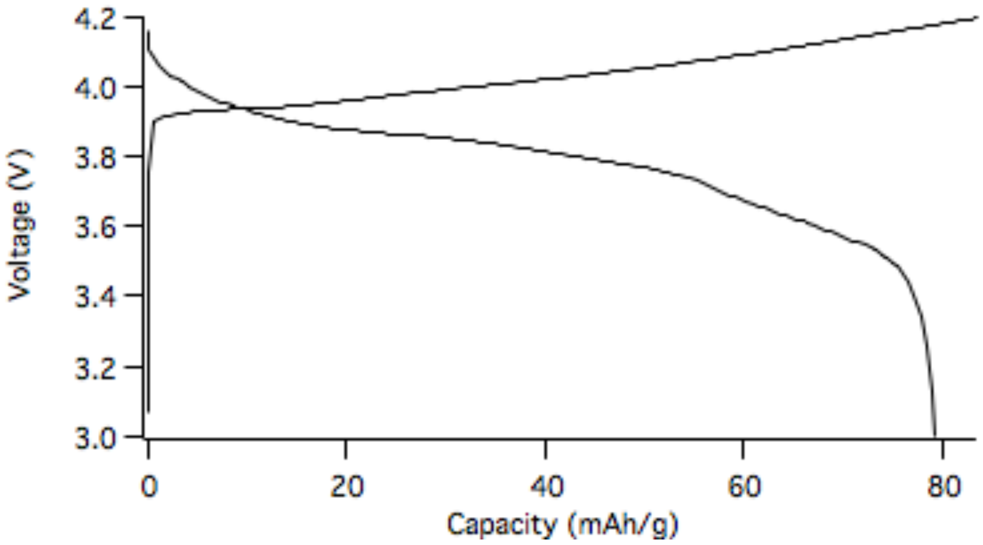

Figure S17. Charge/discharge profile of $LiCoO_2$/hPNB/Li battery at a discharge rate of 0.5 C.

References:

1. R. Khurana, J. L. Schaefer, L. A. Archer and G. W. Coates, *J Am Chem Soc*, 2014, **136**, 7395–7402.
2. J. M. Blanco, F. Fernández, X. García-Mera and J. E. Rodríguez-Borges, *Tetrahedron*, 2002, **58**, 8843–8849.
3. P. Bruce, *Solid State Ionics*, 1988, **28-30**, 918–922.
